# Supplementary material for: The isochromosome 20q abnormality of pluripotent cells interrupts germ layer differentiation
Source: Stem Cell Reports. 2023 Feb 16;18(3):782–97. doi: 10.1016/j.stemcr.2023.01.007 (PMC10031278; doi:10.1016/j.stemcr.2023.01.007)
Supplement: Document S1. Figures S1–S7, Table S1, and supplemental experimental procedures [file mmc1.pdf]

Stem Cell Reports, Volume *18*

Supplemental Information

The isochromosome 20q abnormality of pluripotent cells interrupts germ layer differentiation

Loriana Vitillo, Fabiha Anjum, Zoe Hewitt, Dylan Stavish, Owen Laing, Duncan Baker, Ivana Barbaric, and Pete Coffey

FIGURE S1
Related to FIGURE 1

A

Clone	Karyotype
M7 A1	A46,XY [20]
M7 A2	A46,XY [20]
M7 A5	A46,XY,i(20)(q10) [20]
M7 A9	A46,XY,i(20)(q10) [20]
M8 WT	A46,XY [30]
M8 iso20q	A46,XY,i(20)(q10) [20]
M13 WT	A46,XY [30]
M12 iso20q	A46,XY,i(20)(q10) [20]

B

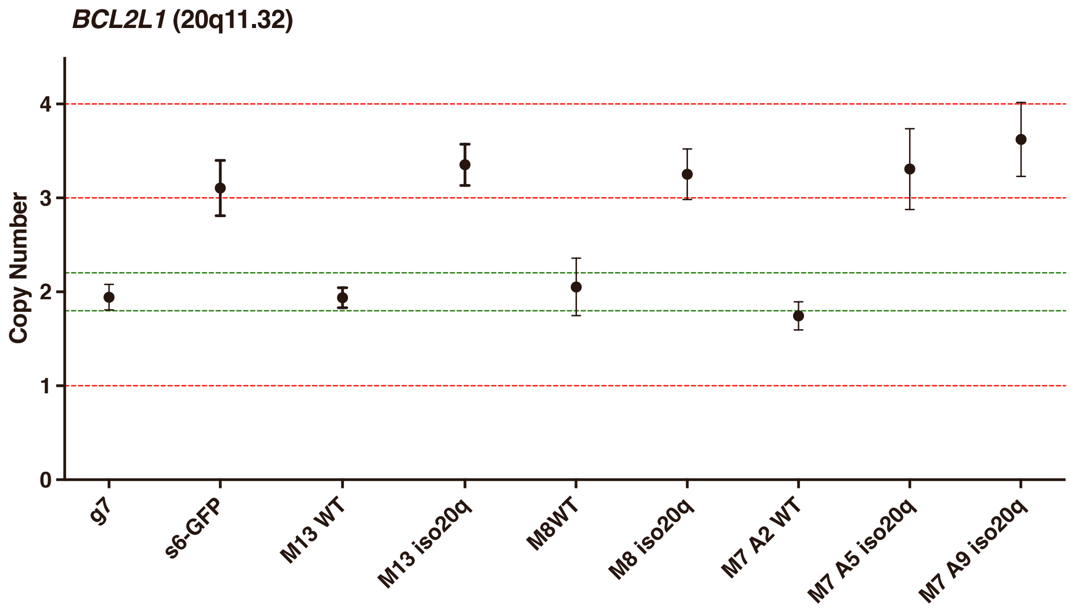

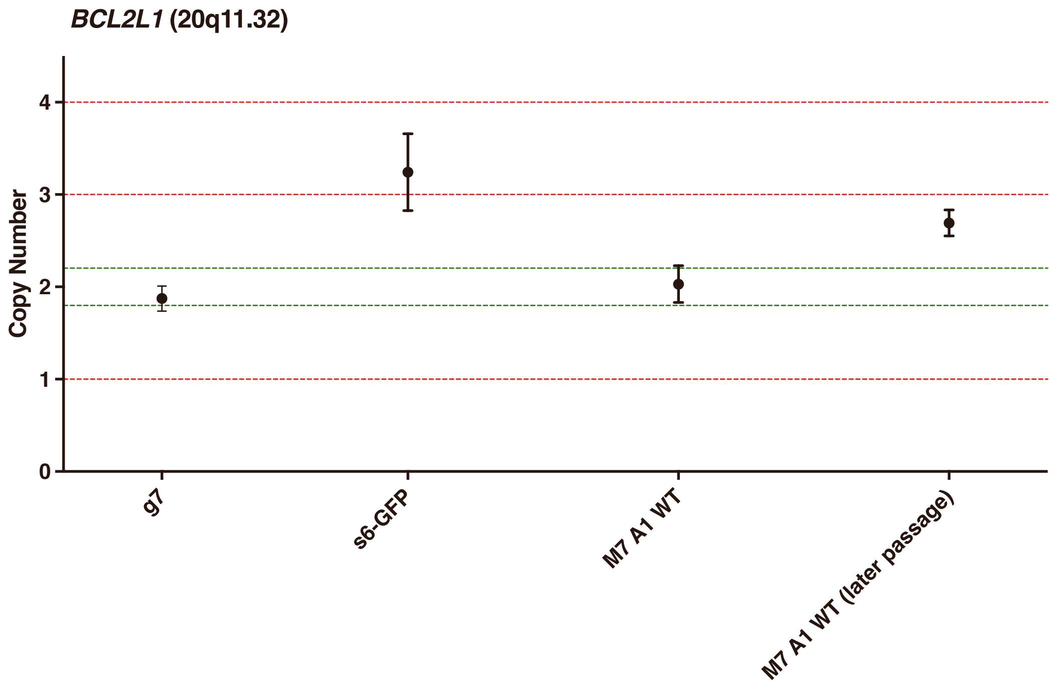

FIGURE S2
Related to FIGURE 2

A

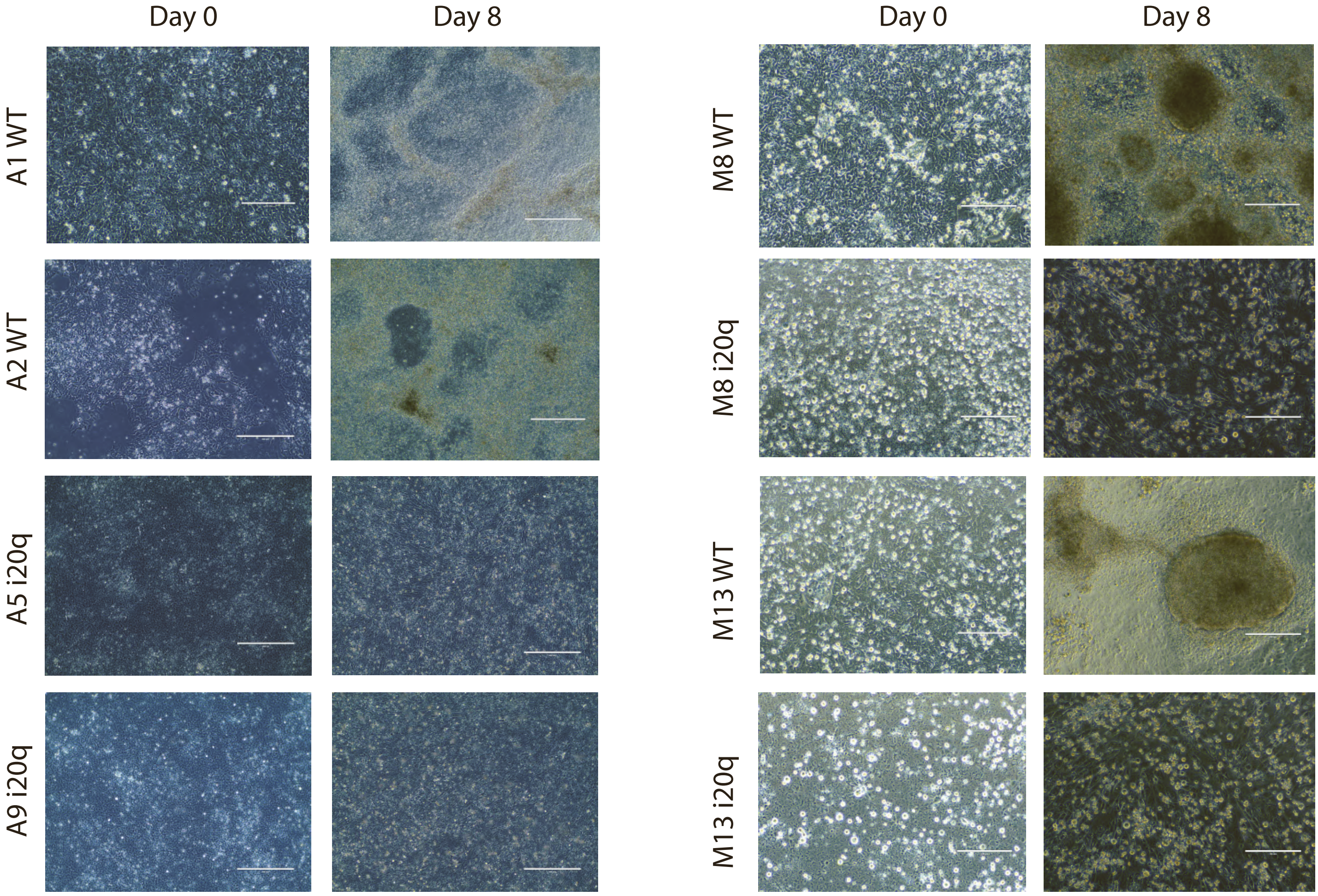

B

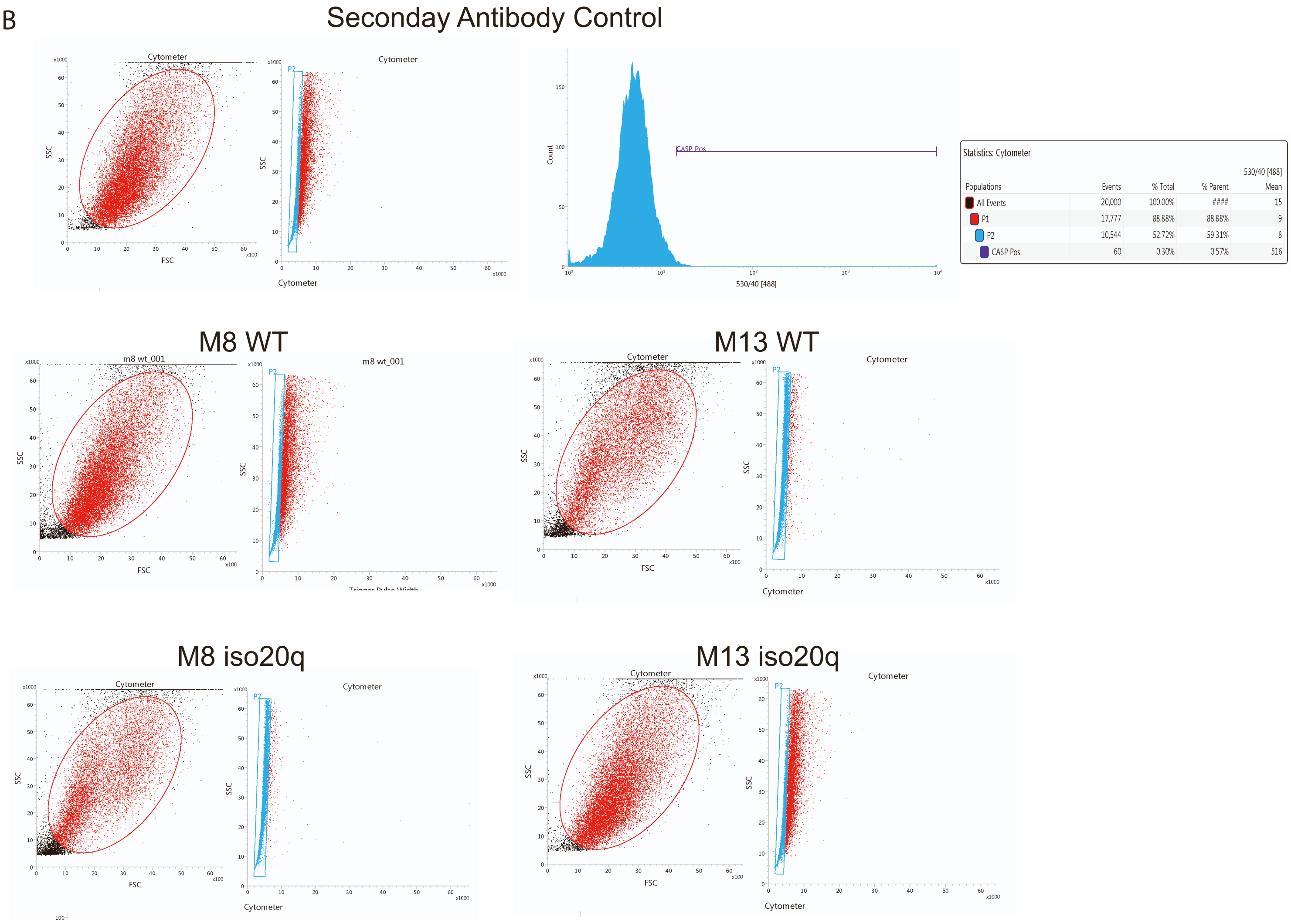

Statistics: Cytometer

Populations	Events	% Total	% Parent	530/40 [488] Mean
All Events	20,000	100.00%	####	15
P1	17,777	88.88%	88.88%	9
P2	10,544	52.72%	59.31%	8
CASP Pos	60	0.30%	0.57%	516

FIGURE S3
Related to FIGURE 3

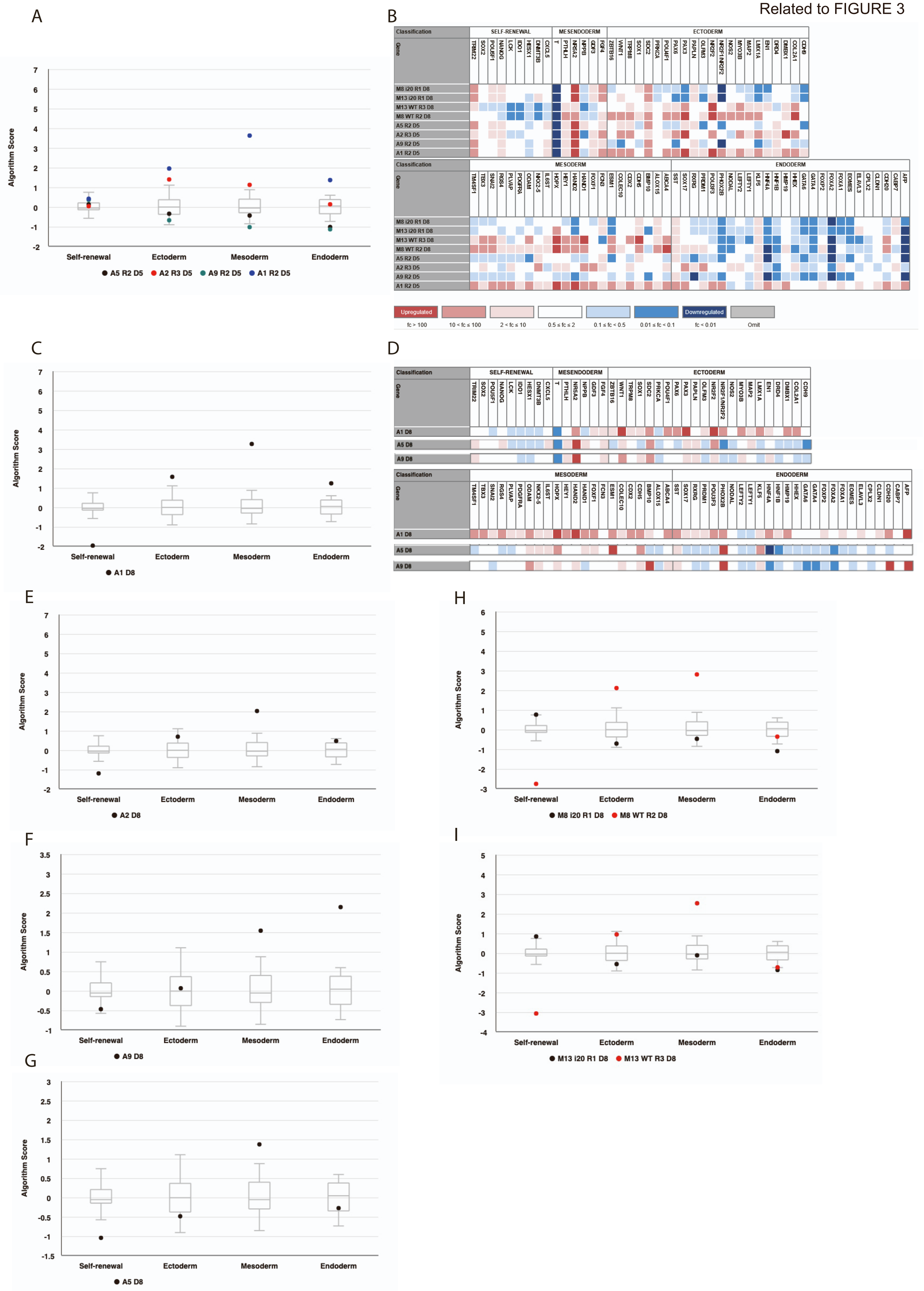

FIGURE S4
Related to FIGURE 3

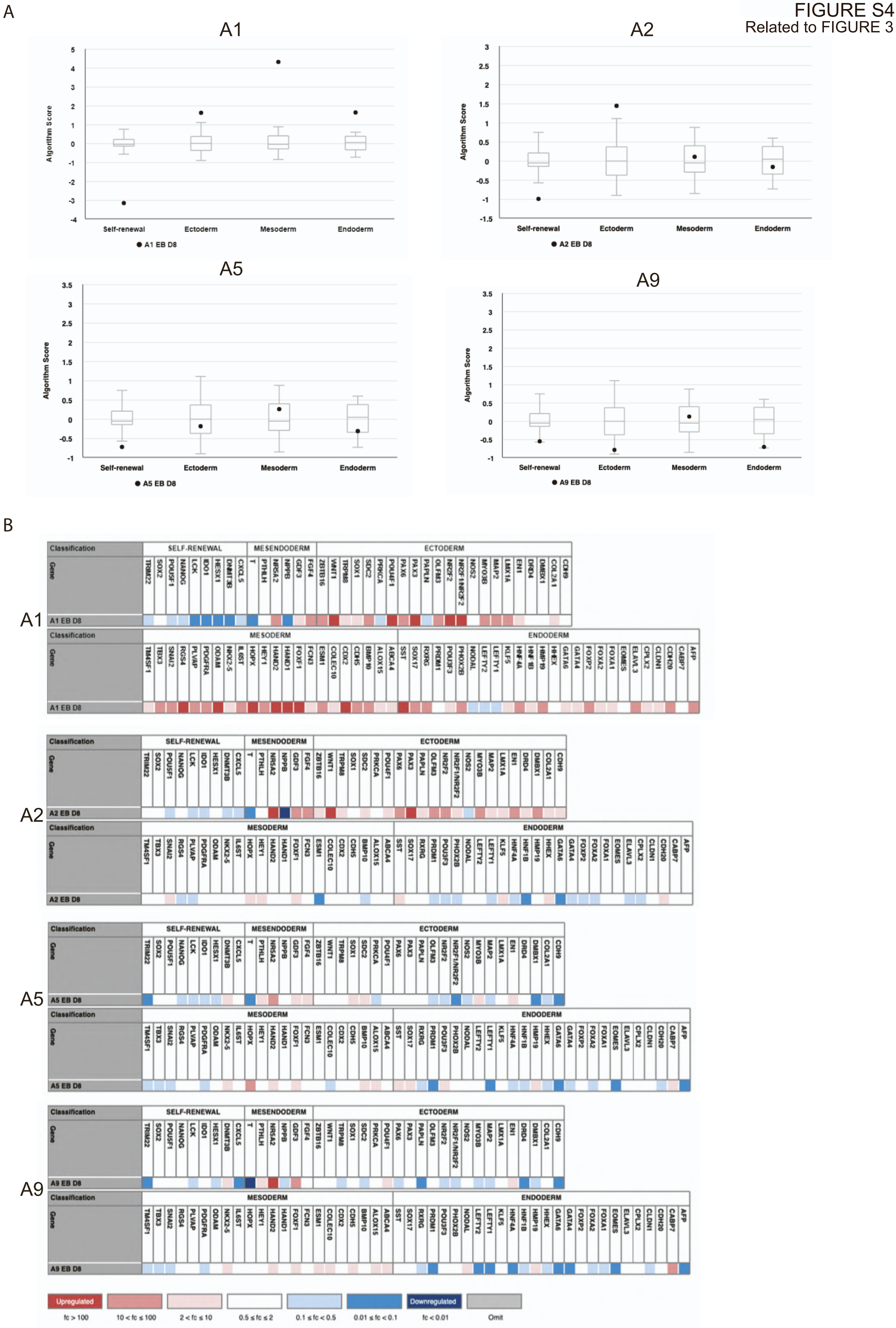

FIGURE S5
Related to FIGURE 4/5

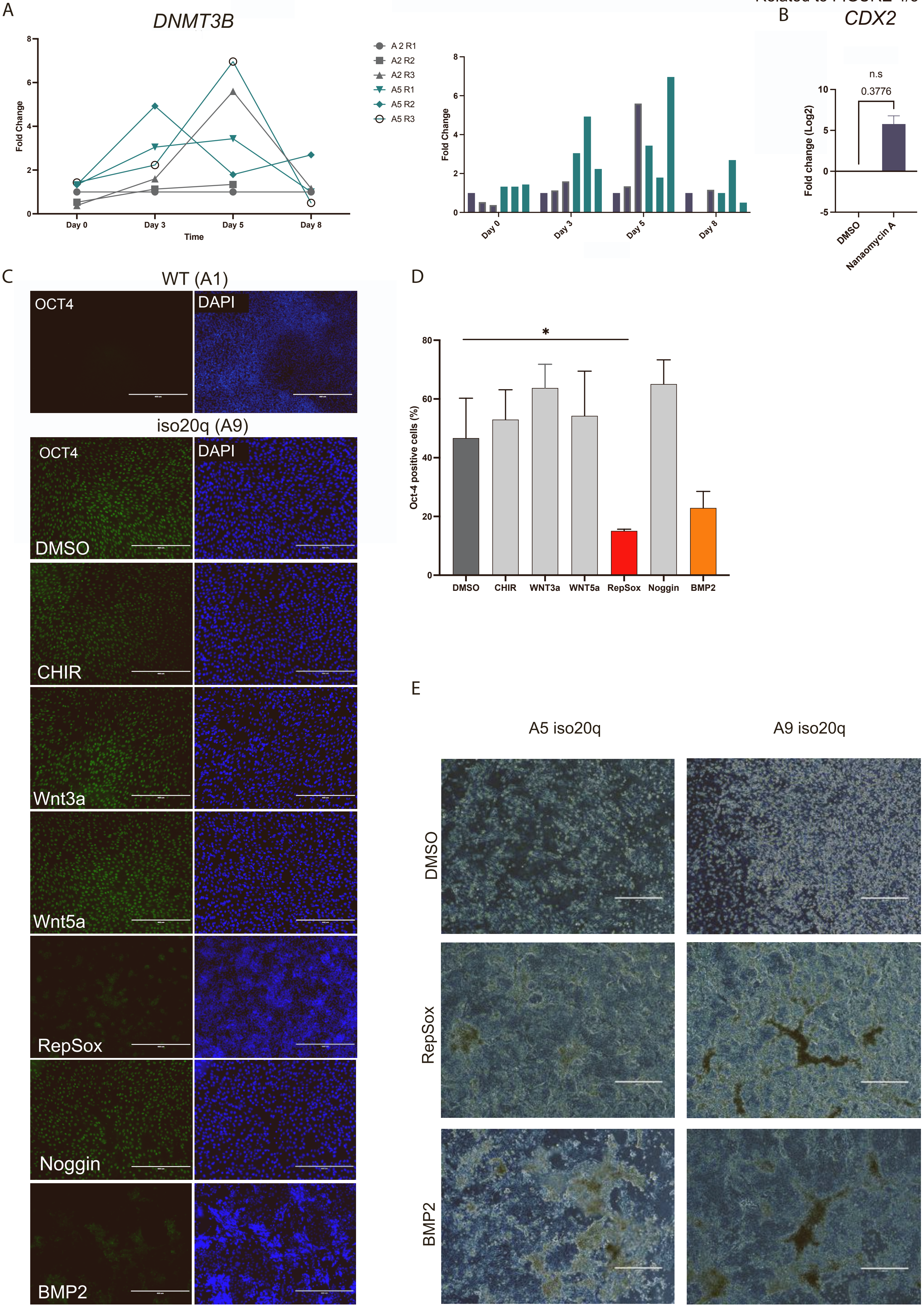

A

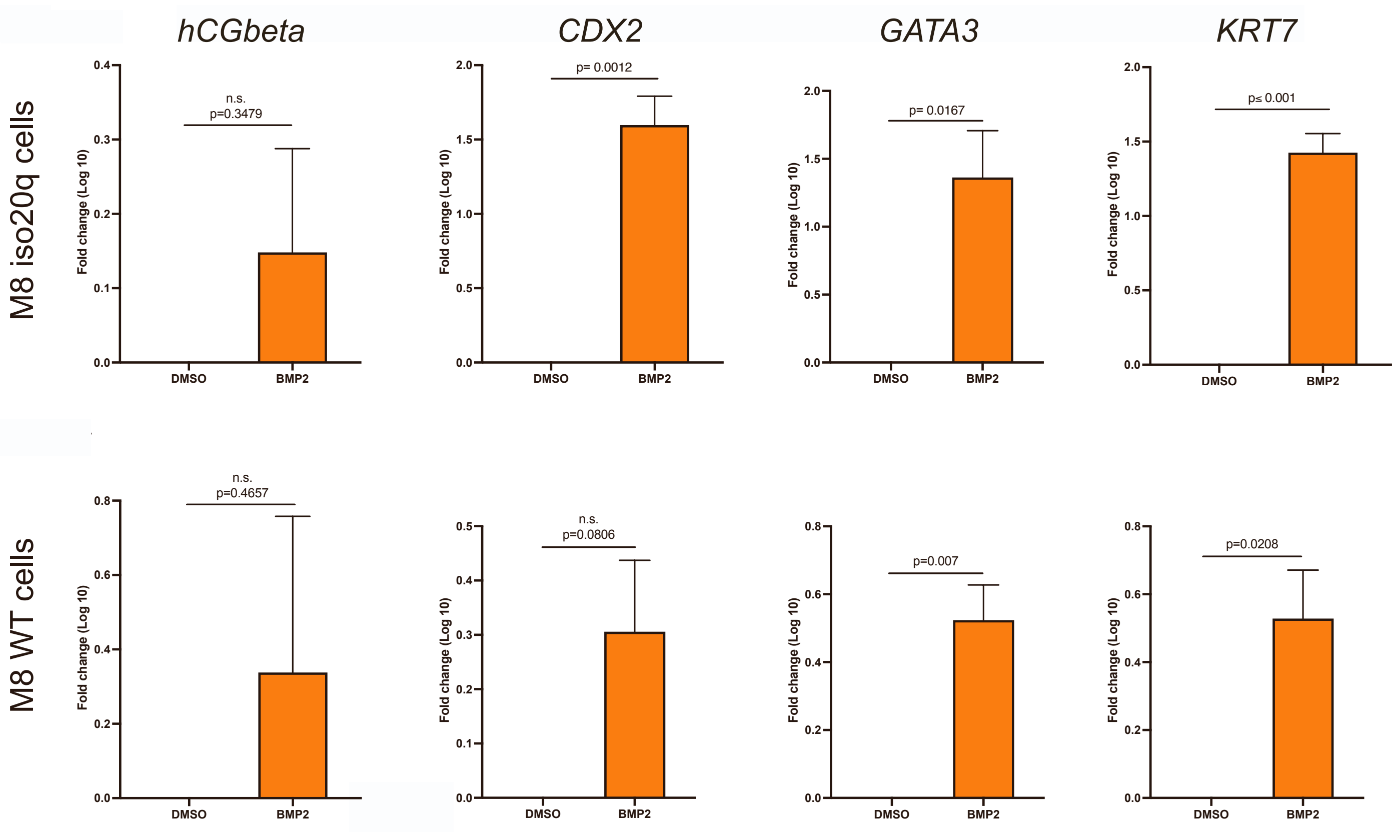
hCGbeta
CDX2
GATA3
KRT7
M8 iso20q cells
M8 WT cells
Fold change (Log 10)
DMSO
BMP2
n.s.
p=0.3479
p= 0.0012
p= 0.0167
p≤ 0.001
p=0.4657
p=0.0806
p=0.007
p=0.0208

B

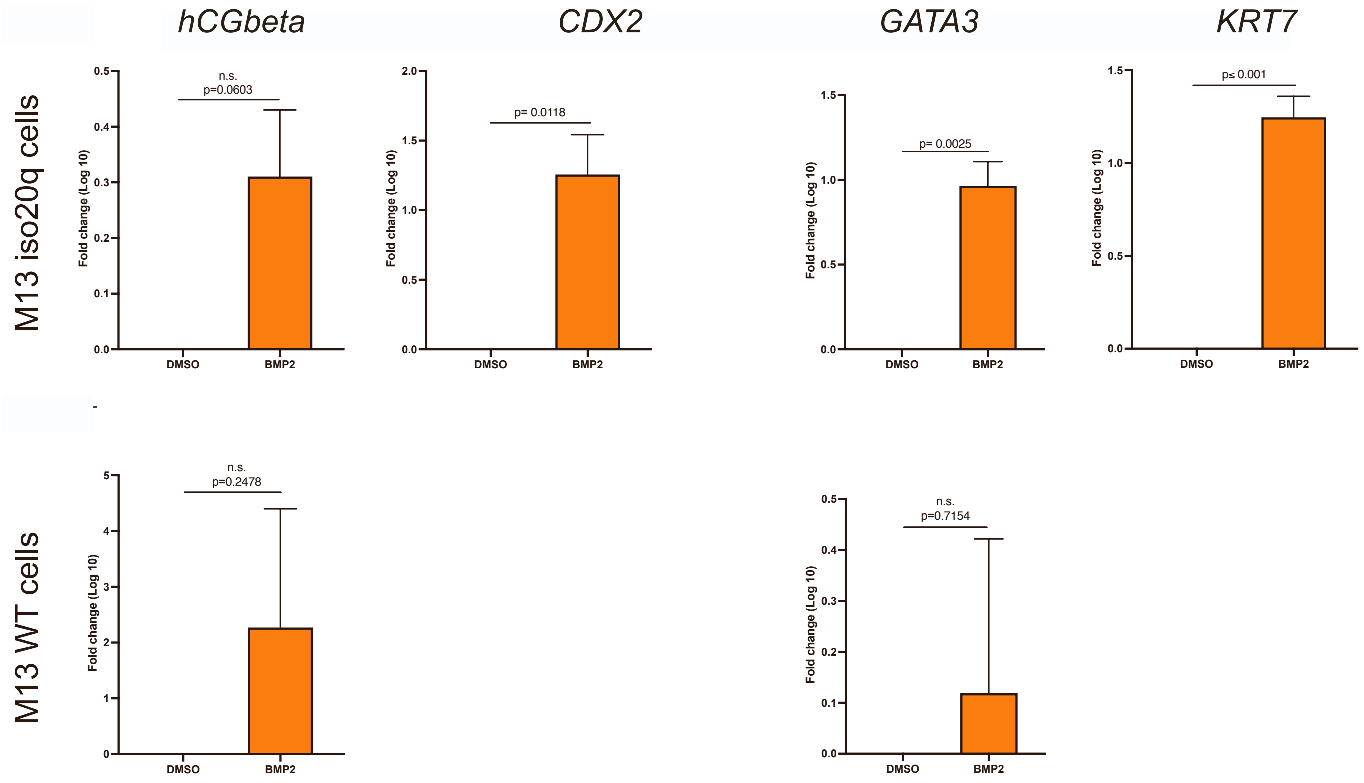
hCGbeta
CDX2
GATA3
KRT7
M13 iso20q cells
M13 WT cells
Fold change (Log 10)
DMSO
BMP2
n.s.
p=0.0603
p= 0.0118
p= 0.0025
p≤ 0.001
p=0.2478
p=0.7154

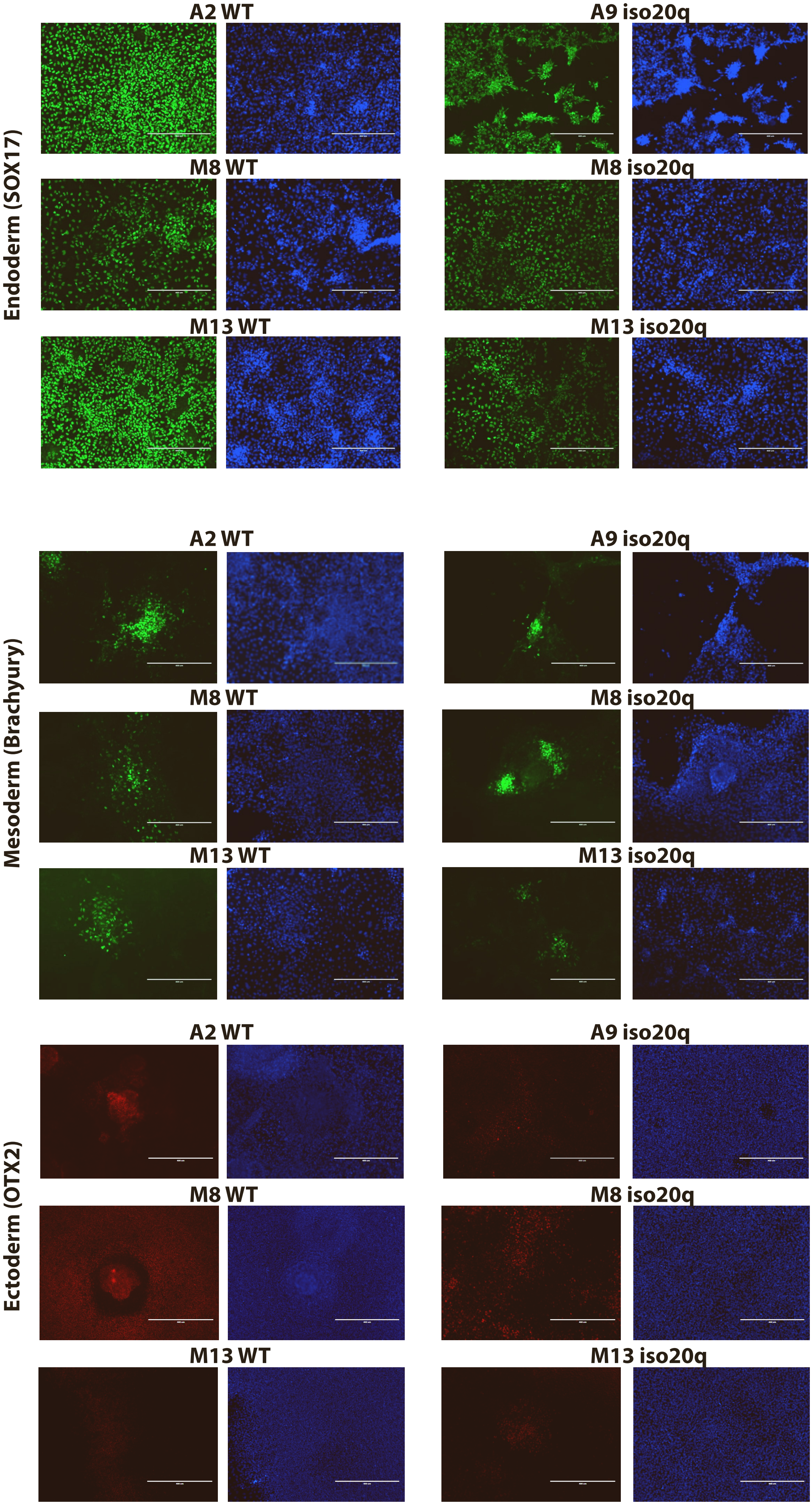
Endoderm (SOX17)
A2 WT
A9 iso20q
M8 WT
M8 iso20q
M13 WT
M13 iso20q
Mesoderm (Brachyury)
A2 WT
A9 iso20q
M8 WT
M8 iso20q
M13 WT
M13 iso20q
Ectoderm (OTX2)
A2 WT
A9 iso20q
M8 WT
M8 iso20q
M13 WT
M13 iso20q

SUPPLEMENTAL FIGURES LEGENDS

Figure S1. Isolating culture acquired chromosome 20 genetic abnormalities.
(Related to Figure 1)

(A) Summary of final quality control karyology for the MasterShef-7, -8, -13 clonal and banked lines.
(B) qPCR copy number assay for BCL2L1 (20q.11.32 locus) in isolated clonal and banked lines. Negative and positive calibrators g7 and s6-GFP are also shown.

Figure S2. Iso20q variants cannot survive a spontaneous RPE differentiation.
(Related to Figure 2)

(A) Representative phase images of iso20q/WT pairs at day 0 and day 8 of RPE differentiation. Scale bars 200 μm.
(B) Gating strategies utilised in caspase-3 flow cytometry analysis in M8 and M13 isogenic lines. Secondary control illustrates the final population of interest (P2) and signal baseline.

Figure S3. The iso20q variant interrupts germ layer differentiation.
(Related to Figure 3)

(A) Algorithm box plot from scorecard qPCR array in A clones at day 5 of differentiation.
(B) Pluripotency scorecard heatmap in iso20q/WT clonal pairs at day 5 (A1, A2, A5, A9 clones) and day 8 (M8, M13 pairs).
(C) Algorithm box plot from scorecard qPCR array in A1 clone at day 8 of differentiation.
(D) Pluripotency scorecard heatmap in iso20q/WT clonal pairs at day 8 (A1, A2, A5, A9 clones).
(E) Algorithm box plot from scorecard qPCR array in A2 clone at day 8 of differentiation.
(F) Algorithm box plot from scorecard qPCR array in A9 clone at day 8 of differentiation.
(G) Algorithm box plot from scorecard qPCR array in A5 clone at day 8 of differentiation.
(H) Algorithm box plot from scorecard qPCR array in M8 WT and iso20q clones at day 8 of differentiation.
(I) Algorithm box plot from scorecard qPCR array in M13 WT and iso20q clones at day 8 of differentiation.

Figure S4. The iso20q variant interrupts germ layer differentiation.
(Related to Figure 3)

(A) Algorithm box plots from scorecard qPCR array in A1, A2, A5, A9 clones at day 8 of EBs formation.
(B) Pluripotency scorecard heatmap in A1, A2, A5, A9 clones at day 8 EBs formation.

Figure S5. DNMT3B-overexpressing iso20q variants are not nullipotent. BMP2 and TGFβ inhibition induce differentiation of iso20q cells.
(Related to Figure 4 and 5)

(A) DNMT3B gene expression in iso20q A5 versus normal A2 clones during 8 days of spontaneous differentiation. R represents an individual experimental run.
(B) Gene expression of trophectoderm marker CDX2 in WT A2 at day 8 of differentiation following treatment with DMSO or nanaomycin A. Data are presented as mean plus SEM, and statistical significance was determined using Student’s t test, two-tailed, n=3; ns, not significant.
(C) Representative immunofluorescence images of iso20q A9 clones treated with DMSO or CHIR99021 (1μM), Wnt3A (100 ng/mL), Wnt5A (100 ng/mL), RepSox (100 nM), Nogging (200 ng/mL) or BMP2 (100 ng/mL) for 8 days of spontaneous RPE differentiation and stained with OCT4 and DAPI. The WT A1 clone treated with DMSO is shown as control. Scale bar 400μm.
(D) Quantification of OCT4-positive cells in iso20q A9 clones treated with DMSO or CHIR99021 (1μM), Wnt3A (100 ng/mL), Wnt5A (100 ng/mL), RepSox (100 nM), Nogging (200 ng/mL) or BMP2 (100 ng/mL) for 8 days of RPE differentiation. Data are presented as mean plus SEM, and statistical significance was determined using Student’s t test, one-tailed; n=3 independent experiments.
(E) Representative phase images showing the morphological change of A5 and A9 iso20q clones treated with 100 nM RepSox or 100 ng/mL BMP2 versus DMSO for 8 days of RPE differentiation. Scale bars 400μ.

Figure S6. The iso20q variant retain differentiation competency towards extra-embryonic amnion. (Related to Figure 6)

(A) Gene expression of trophoblast markers in M8 iso20q and M8 WT treated with 100 ng/mL BMP2 for 4 days of the RPE differentiation. Data are presented as mean plus SEM, and statistical significance was determined using Student's t test, one-tailed; n=3; n.s. not significant.

(B) Gene expression of trophoblast markers in M13 iso20q and M13 WT treated with 100 ng/mL BMP2 for 4 days of the RPE differentiation. Data are presented as mean plus SEM, and statistical significance was determined using Student's t test, one-tailed; n=3; n.s. not significant.

Figure S7. Directed differentiation protocols mask iso20q underlying developmental defect

(A) Representative immunofluorescence images of iso20q and WT isogenic lines after directed differentiation protocols for endoderm (SOX17), mesoderm (Brachyury) and ectoderm (OTX2). DAPI counterstains are shown in parallel. Scale bars 400μm.

SUPPLEMENTAL EXPERIMENTAL PROCEDURES

Single cell cloning.

Cloning was performed by single cell deposition using a BD FACSJazz cell sorter (BD Biosciences) into 96 well plates with human feeder cells, NutriStem hPSC XF medium (Biological Industries) supplemented with Rock inhibitor Y-27632 (Generon, A11001). After 24 hours the medium was changed to remove Y-27632 and the clones were expanded for 2 weeks before manual picking and culture as described for research cultures. Seed banks were frozen from each clone after 5 passages and material taken for detection of variants by karyotyping and qPCR. Once the genotypes were confirmed the clonal lines were thawed and expanded. MasterShef-7 A1, A2, A5 and A9, were all cloned from the same heterogenous starting population. MasterShef-7, which was derived directly from clinical material but cultured for 8 passages on laminin-521/NutriStem prior to being frozen at passage P5+8. Having indicated a heterogenous i20q karyotype at ~70% prior to freeze, single cell cloning was performed at passage 2 after recovery (P5+8+2) to obtain matched wild type cells from the same culture. Note: a '+' in the passage number refers to a thaw from a cell bank. MasterShef-8 WT (Clone C1.1) was cloned from a homogenous cell bank derived directly from the clinical material but cultured for 5 passage on laminin-521 in NutriStem prior to being frozen P2+9+5 and then subsequently thawed for cloning. The i20q variant (Clone C5.1) was cloned from separate research material which had been culture under its derivation conditions, and then Cellstart/NutriStem before being switched (at P2+9+5+9) to laminin-521/NutriStem culture and frozen as MasterShef-8 QC2.1 at passage P2+9+5+9+19 having acquired the variant which at the point of freeze accounted for ~90% of the cells present. MasterShef-13 WT and i20q lines, were not clonal lines, coming from different research cell banks. Wild type cells came from MasterShef-13 QC1.4 P6+3 derived directly from the clinical material, while MasterShef-13 i20q cells came from bank QC3.1 P6+3+19 cultured on laminin-521.

Genetic copy number q-PCR assay.

Quantitative PCR detection of copy number at loci 20q was performed with our previously described methods and primers[1].

Flow cytometry.

Suspended apoptotic cells were collected together with adherent cells harvested with TrypLE (Thermo Fisher Scientific, 11528856). The collated sample was pelleted and fixed in 4% PFA at room temperature. Flow cytometry for cleaved caspase-3 (Cell Signalling, 9661) was performed with the same methods and reagents we described previously[2]. Samples were analysed using a BD FACSJazz (BD Biosciences). The signal of secondary antibody-only stained samples was used to determine the level of baseline fluorescence. Gating strategies are shown in Figure S2.

Embryoid Bodies.

Confluent hESCs were passaged 1 to 1 into a low-adherence square well plate in 50% Nutristem 50 % TLP media plus 1:1000 Rock inhibitor Y-27632. After 24 hours the newly formed aggregates were transferred to a 15 ml tube to settle and media was fully replaced to TLP. Media was replaced every other day for a week at which point embryoid bodies were collected for RNA extraction.

Immunofluorescence.
Cells were fixed at room temperature for 30 minutes in 4% paraformaldehyde followed by three washes in DPBS. Blocking and permeabilization was performed in DPBS plus 5% Normal Donkey Serum (Jackson Immuno Research Laboratories, 017-000-121) in 0.3% triton X-100 (Sigma). Cells were incubated overnight at 4°C with primary antibodies diluted in 1% donkey serum, 0.3% triton X-100. Next, cells were washed 3 times with DPBS and incubated for 2 hours at room temperature with fluorescent FITC- or TRITC-conjugated donkey secondary antibodies against IgG species matching (Jackson Immuno Research Laboratories) at a dilution of 1:100 in 2% donkey serum, 0-3% triton X-100 (in DPBS). Cells were washed 3 times with DPBS. For nuclei counterstain, cells were incubated for 60 seconds with DAPI (Sigma, D9542, 1:2500) followed by another 3 washes in DPBS. Primary antibodies and dilutions were as follows: goat anti OTX2 (Santa Cruz, 30659, 1:100); rabbit anti OCT-4 (Cell signalling, C30A3, 1:400); goat anti Brachyury (R&D, 967332, 10μg/ml; goat anti SOX17 (R&D, 967330, 10μg/ml). Imaged were acquired using an EVOS FL microscope (Life Technologies) and analysed using Cell Profiler software.

Real-time quantitative PCR.
RNA was extracted from cell pellets using the PureLink RNA mini kit following manufacturer's instruction (Invitrogen). RNA was retrotranscribed to cDNA using the SuperScript III Synthesis System (Invitrogen) or the High capacity cDNA Reverse transcription kit (Applied Biosystems) following manufacturer's instruction. Transcript levels were detected using PowerSYBR™ Green master mix (Applied Byosystems) with StepOne Plus (Applied Biosystems). Gene expression was normalised to GAPDH levels for each sample and final fold change were calculated using the $2^{-\Delta\Delta Ct}$ algorithm. Primers sequence are listed in Table S1.

Western blot array.
Semi-quantitative measurement of 15 stem cell protein markers was performed with the human Stem Cell Array C1 (RayBio®) following manufacturer instructions. Cell pellets were lysed with the supplied lysis buffer supplemented with 1:500 Protease inhibitor cocktail set III (Merk Millipore). Protein extracts were quantified with a Bradford assay (Bio-Rad) and BSA standard curve detected with a Nanodrop™ One (Life Technologies) spectrophotometer. A final amount of 250 μg was loaded onto each protein array. Blots were scanned with Chemidoc (Bio-Rad). Densitometries were extracted from scanned high-resolution images with ImageJ software. Results were analysed according to manufacturer instruction considering averages of target signals relative to internal positive controls and normalised to reference array (DMSO array).

Small molecules and morphogens.
DNMT3b inhibitor Nanaomycin A (BioVision, 2790-1), CHIR990-21 (Miltenyi, StemMACS™), recombinant human Wnt3a (R&D, 5036-WN-010), recombinant human/mouse Wnt5a (R&D, 645-WN-010), RepSox, recombinant human Noggin (R&D, 6057-NG-025), recombinant human/mouse/rat BMP2 (R&D, 355-BM-010), DMSO (Sigma, 02438).

Directed differentiation.
Directed trilineage differentiation were performed with standardised StemXVivo Endoderm (R&D, SC019B), StemXVivo Mesoderm (R&D, SC030B) and STEMdiff™ Trilineage Ectoderm Medium (05231) kits, following manufacturer's instruction. Endoderm and Mesoderm differentiations were stopped at day 4 while ectoderm differentiation was stopped at day 7. For these experiments cells were expanded in mTERS1 (Stem Cell Technologies, 85850) and plated on Cultrex Basement Membrane Extract (R&D, 3432-005-01) for Endoderm and Mesoderm differentiation or Matrix Matrigel hESc qualified (Corning, 734-1440) for Ectoderm differentiation.

Table S1. Primers set.

Gene target	Forward	Reverse
DNMT3B	CTGCCGGTGTTTCTGTGTGG	TGTAACAGCTCCAGGGCTCC
BCL-XL	CTGCCGGTGTTTCTGTGTGG	TCCACAAAAGTATCCCAGCC
OTX2	GCGCAGCTAGATGTGCTGGA	CACTGCTGCTGGCAATGGTC
PAX6	CCATCAGACCCAGGGCAATC	GGTCTGCCCGTTCAACATCC
CDX2	TTCACTACAGTCGCTACATCACC	TTGATTTTCCTCTCCTTTGCTC
hCGB	ACCCTGGCTGTGGAGAAGG	ATGGACTCGAAGCGCACA
GATA3	TGCAGGAGCAGTATCATGAAGCCT	GCATCAAACAACTGTGGCCAGTGA
KRT7	AGGATGTGGATGCTGCCTAC	CACCACAGATGTGTCGGAGA
GAPDH	CCCCACCACACTGAATCTCC	GGTACTTTATTGATGGTACATGACAAG
	**Reference number (Thermo Fisher Scientific)**	
SOX17	HS00751752_s1	
T	Hs00610080_m1	

SUPPLEMENTAL REFERENCES

1) Laing, O., Halliwell, J., and Barbaric, I. (2019). Rapid PCR Assay for Detecting Common Genetic Variants Arising in Human Pluripotent Stem Cell Cultures. Curr Protoc Stem Cell Biol 49, e83. 10.1002/cpsc.83.

2) Price, C.J., Stavish, D., Gokhale, P.J., Stevenson, B.A., Sargeant, S., Lacey, J., Rodriguez, T.A., and Barbaric, I. (2021). Genetically variant human pluripotent stem cells selectively eliminate wild-type counterparts through YAP-mediated cell competition. Dev Cell 56, 2455-2470 e2410. 10.1016/j.devcel.2021.07.019.
