# Supplementary material for: The isochromosome 20q abnormality of pluripotent cells interrupts germ layer differentiation
Source: Stem Cell Reports. 2023 Feb 16;18(3):782–97. doi: 10.1016/j.stemcr.2023.01.007 (PMC10031278; doi:10.1016/j.stemcr.2023.01.007)
Supplement: Document S1. Article plus supplemental information [file mmc2.pdf]

Stem Cell Reports
Article
OPEN ACCESS

The isochromosome 20q abnormality of pluripotent cells interrupts germ layer differentiation

Loriana Vitillo,[1,*] Fabiha Anjum,[1] Zoe Hewitt,[2] Dylan Stavish,[2] Owen Laing,[2] Duncan Baker,[3] Ivana Barbaric,[2] and Pete Coffey[1,4,5]
[1]Rescue, Repair and Regeneration, Institute of Ophthalmology, University College London, EC1V 9EL London, UK
[2]Centre for Stem Cell Biology, School of Biosciences, University of Sheffield, S10 2TN Sheffield, UK
[3]Sheffield Diagnostic Genetic Services, Sheffield Children's Hospital, Sheffield, UK
[4]Centre for Stem Cell Biology and Engineering, University of California, Santa Barbara, Santa Barbara, CA, USA
[5]NIHR Biomedical Research Centre at Moorfields Eye Hospital NHS Foundation Trust, UCL Institute of Ophthalmology, London, UK
*Correspondence: l.vitillo@ucl.ac.uk
https://doi.org/10.1016/j.stemcr.2023.01.007

ASSOCIATED PODCAST For an associated discussion of this work, listen to the latest episode of *The Stem Cell Report* podcast at https://www.isscr.org/podcast/s2-e8, brought to you by the ISSCR.

SUMMARY

Chromosome 20 abnormalities are some of the most frequent genomic changes acquired by human pluripotent stem cell (hPSC) cultures worldwide. Yet their effects on differentiation remain largely unexplored. We investigated a recurrent abnormality also found on amniocentesis, the isochromosome 20q (iso20q), during a clinical retinal pigment epithelium differentiation. Here we show that the iso20q abnormality interrupts spontaneous embryonic lineage specification. Isogenic lines revealed that under conditions that promote the spontaneous differentiation of wild-type hPSCs, the iso20q variants fail to differentiate into primitive germ layers and to downregulate pluripotency networks, resulting in apoptosis. Instead, iso20q cells are highly biased for extra-embryonic/amnion differentiation following inhibition of DNMT3B methylation or BMP2 treatment. Finally, directed differentiation protocols can overcome the iso20q block. Our findings reveal in iso20q a chromosomal abnormality that impairs the developmental competency of hPSCs toward germ layers but not amnion, which models embryonic developmental bottlenecks in the presence of aberrations.

INTRODUCTION

The keystone for the biomedical applications of human pluripotent stem cells (hPSCs) is their unlimited self-renewal in culture. However, hPSCs have the propensity to acquire genetic abnormalities that confer fitness for prolonged expansion and that could sabotage research interpretation and clinical studies (Andrews et al., 2017; Halliwell et al., 2020; Sato et al., 2019). The most common and recurrent hPSC abnormalities involve whole or partial gains on chromosomes 1, 12, 17, 20, and X (Baker et al., 2016; International Stem Cell Initiative et al., 2011; Lund et al., 2012). Strikingly, chromosome 20q aberrations have been found in almost 20% of hPSCs tested worldwide (Avery et al., 2013; International Stem Cell Initiative et al., 2011).

Several studies have reported on the origin of hPSCs aneuploidies and on the mechanisms of selective advantage in culture, such as faster cell cycle and escape from apoptosis (Avery et al., 2013; Halliwell et al., 2020; Olariu et al., 2010; Taapken et al., 2011; Thompson et al., 2020). Recently, we showed that during self-renewal abnormal cells prevail over wild-type (WT) cells via mechanically dependent cell competition (Price et al., 2021). However, the functional effects of most genetic abnormalities during differentiation remain poorly understood. Reports available point to mixed phenotypes, including reduced, delayed or alternative differentiation capacity *in vitro* and immature teratomas *in vivo* (Ben-David et al., 2014; Fazeli et al., 2011; Keller et al., 2018; Lee et al., 2015; Markouli et al., 2019, 2021; Werbowetski-Ogilvie et al., 2009). Yet from the studies available, it is becoming evident that the impact of hPSC abnormalities on differentiation is lineage specific and protocol dependent. This is particularly poignant for hPSCs derivatives used in regenerative medicine, as undetected abnormalities, especially if cancer related, could derail disease modeling and clinical trials (Garber, 2015; Sato et al., 2019).

The retinal pigment epithelium (RPE) is a major disease-relevant cell type in hPSC-based regenerative medicine (Kobold et al., 2020; Maeda et al., 2021). hPSC-derived RPE cells are in clinical trials for the treatment of age-related macular degeneration, a leading cause of blindness

This is an open access article under the CC BY license (http://creativecommons.org/licenses/by/4.0/).

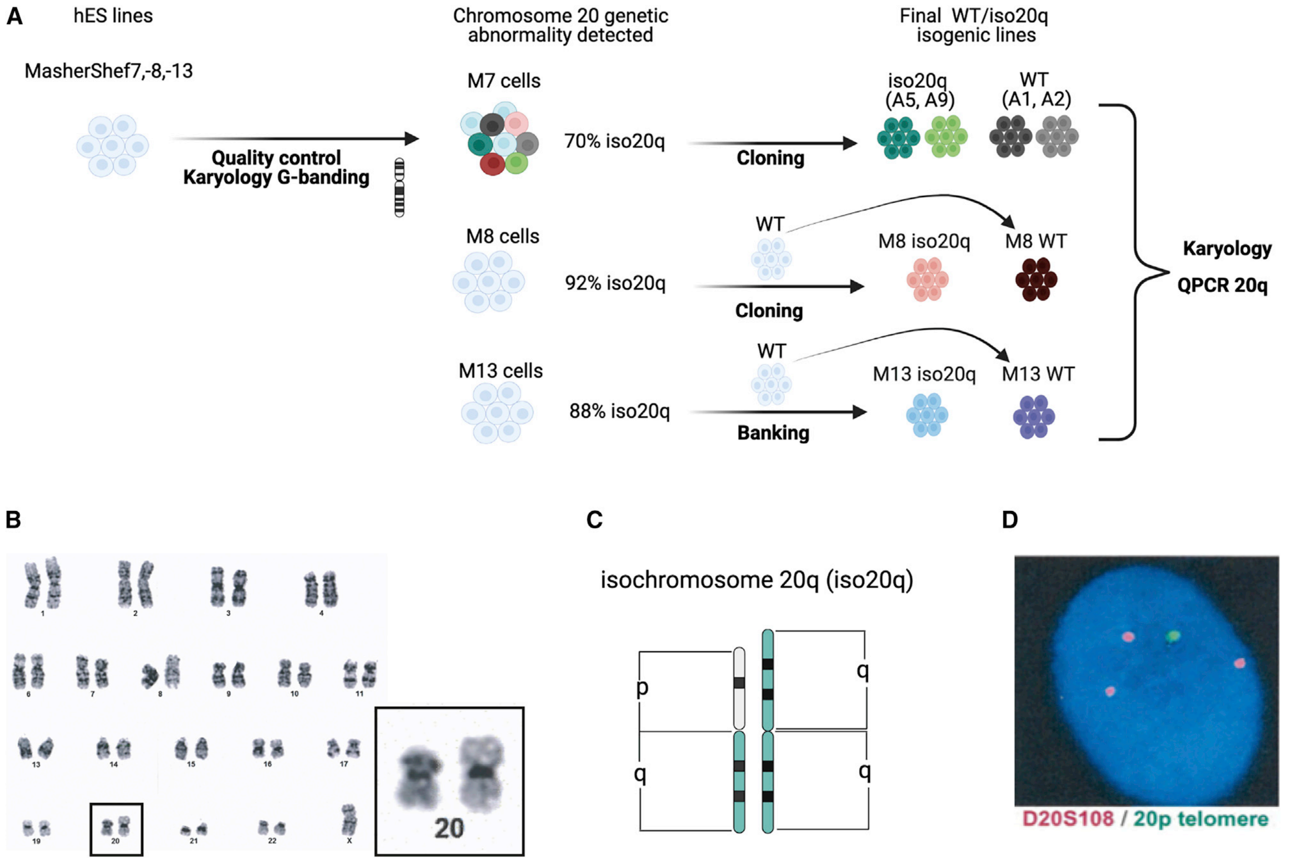

Figure 1. Isolating culture acquired chromosome 20 genetic abnormalities

(A) Diagram of MasterShef-7, MasterShef-8, and MasterShef-13 cell line history from first detection of a karyotype abnormality to isolation and qualification of clonal/homogeneous isogenic WT and iso20q lines.
(B) Bulk karyology of M7 line showing the isochromosome 20q abnormality.
(C) Schematic of the isochromosome 20q variant (iso20q).
(D) FISH analysis of bulk M7 line showing the isochromosome 20q. Telomere probes show monosomy for short arm (20p) and trisomy for long arm (D20S108; 20q).
See also Figure S1.

(Vitillo et al., 2020). However, genomic instability is already a pressing issue in this nascent field of cell therapy. The first in human clinical trial based on induced pluripotent stem cells-derived RPE was halted, and later resumed, because of the discovery of potentially tumorigenic copy number deletions in the stem cells (Garber, 2015; Mandai et al., 2017).

To the best of our knowledge no study has systematically evaluated the consequences of common hPSCs genetic abnormalities for the RPE lineage. Here we investigated the functional effects of a common, but unexplored, culture acquired chromosomal abnormality also detected in human amniocentesis, the isochromosome 20q (Receveur et al., 2017), during a hPSC-RPE differentiation currently in clinical trials (da Cruz et al., 2018).

RESULTS

Isolating culture acquired chromosome 20 genetic abnormalities

Through routine cytogenetic screening, we identified human embryonic stem cell (hESC) cultures of MasterShef-7 (M7), MasterShef-8 (M8), and MasterShef-13 (M13) that had acquired an abnormal karyology while in culture (Figure 1A). Cytogenetic analysis detected the presence of an isochromosome of the long arm of chromosome 20 (iso20q) (Figures 1B and 1C). The iso20q abnormality was also visible by fluorescence *in situ* hybridization (FISH) as consisting of a trisomy for the long arm (20q) and monosomy of the short arm (20p) (Figure 1D). Notably, this abnormality is becoming increasingly common in our

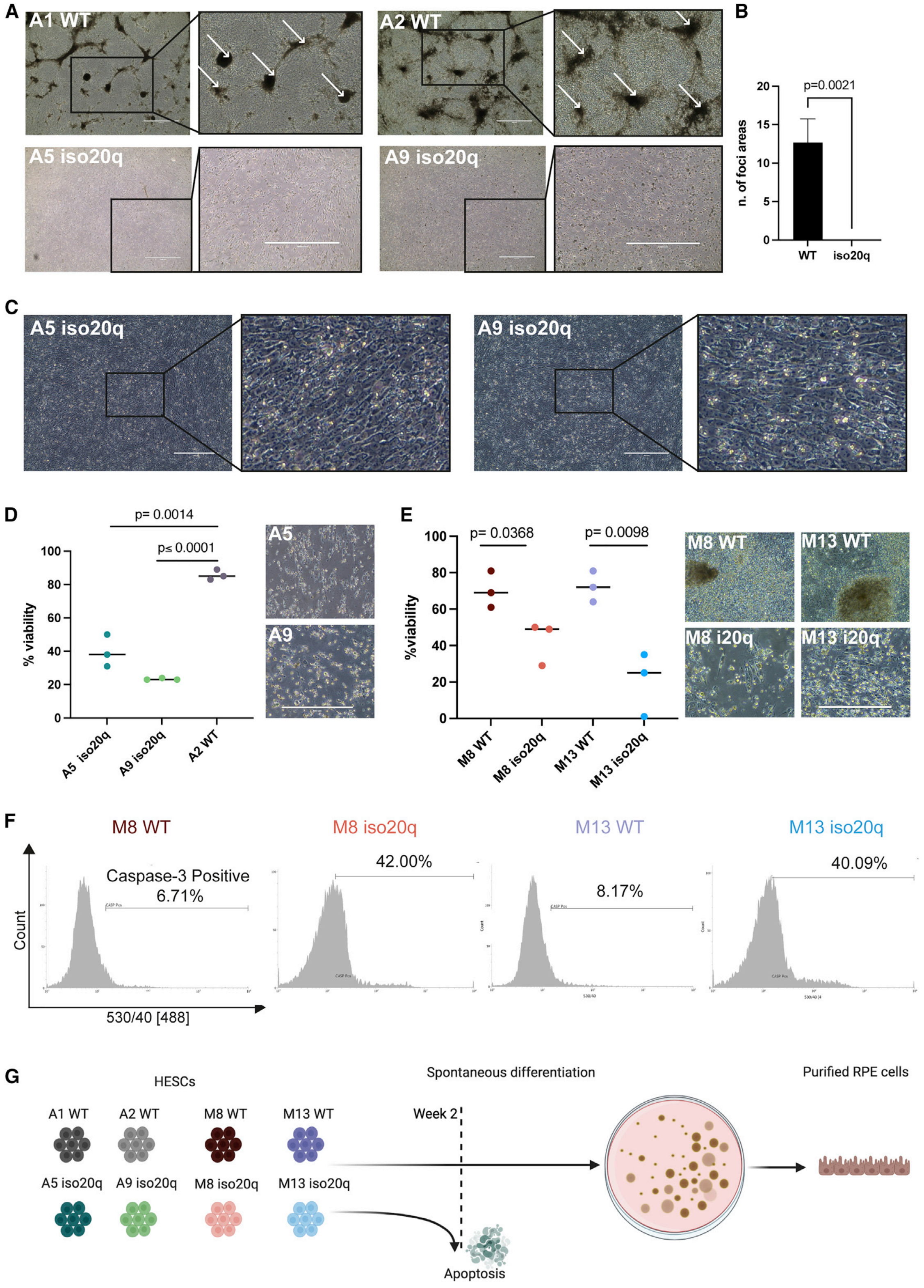

Figure 2. Iso20q variants cannot survive a spontaneous RPE differentiation

(A) Phase images showing morphological differentiation and foci (insert arrows) at day 16 of RPE differentiation in WT but not iso20q variants. Scale bar, 1000 μm.

(legend continued on next page)

karyotyping screenings of hESCs cultured in feeder-free systems. The proportion of cells within each culture displaying the abnormality varied between lines. For MasterShef-8 and MasterShef-13 cultures, karyology showed a near homogeneous population that had acquired the iso20q (92% and 88%, respectively). However, for MasterShef-7, 30% of the population remained karyotypically normal (Figure 1A). Therefore, we derived isogenic clonal lines from M7 with or without iso20q by performing single cell cloning (Figure 1A). We banked isogenic clones of M7; two wild-type lines (A1 and A2) and two iso20q variant lines (A5 and A9) (Figures 1A and S1A). All M7 clones were analyzed using qPCR for the presence of the sub-karyotype 20q11.21 amplicon, which includes the BCL2L1 gene, as it is common among hESC cultures (International Stem Cell Initiative et al., 2011), confirming that WT clones were free of the amplicon, although one clone (A1) acquired it at later passages (Figure S1B). We repeated single cell cloning for the M8 cell line (Figure 1A). However, clones with a normal karyotype were not obtained from within the culture displaying the i20q variant, because the abnormal cells became dominant. Therefore, we generated WT clones from a related bank. The resulting clones were also karyotyped and assessed using qPCR for the presence of BCL2L1 amplicon (Figures S1A and S1B). Given the speed of the overtake of i20q in culture, M13 was not cloned, and instead was used as a representation of a naive bulk near homogeneous population as it is selected for in culture (Figure 1A). The M13 cell bank was shown to be karyotypically normal at an early passage, while iso20q M13 was banked at a later passage after having acquired the variant in almost 90% of the culture. Both M13 WT and M13 iso20q cell banks were thawed and tested immediately prior to inclusion in this study using karyotype and qPCR for the BCL2L1 amplicon (Figures S1A and S1B). Overall, we detected, cloned, qualified and banked iso20q variants that spontaneously emerge in hPSCs and their isogenic wild-type counterparts to use in this and subsequent studies.

Iso20q variants cannot survive a spontaneous RPE differentiation

To assess the behavior of the iso20q abnormality during RPE differentiation, we comparatively ran all the variant pairs in our spontaneous protocol on the basis of FGF2 withdrawal from the self-renewing stem cell media (da Cruz et al., 2018; Vugler et al., 2008). After 1 week, differentiation was visible in WT cells but not in any of the iso20q cell lines and clones (Figure S2A). Two weeks into the protocol, future RPE foci areas were emerging significantly in wild-type but not in iso20q clones (Figures 2A and 2B). Differentiation hallmarks (i.e., areas showing aggregates of cells) were totally absent in the iso20q clones, while cell death was evident (Figure 2A). Strikingly, the iso20q clones displayed a flattened, homogeneous and elongated undifferentiated morphology until around day 10 of the differentiation (Figure 2C). Eventually, iso20q variants died by detachment starting from day 11 halting any further progression in the differentiation beyond two weeks (Figures 2D and 2E). The cell death observed in iso20q cells could be due to programmed cell death mechanisms or uncontrolled necrosis. Therefore, to qualify the type of cell death induced in iso20q variants we quantified the expression of caspase-3, a marker of apoptosis. Flow cytometry revealed a ∼40% increase in caspase-3-positive cells in iso20q cells compared with WT, showing that apoptotic mechanisms underlie the observed cell death (Figures 2F and S2B). In conclusion, our data reveal a unique phenotype for the iso20q variants characterized by a strong resistance to spontaneous differentiation followed by apoptosis of the culture (Figure 2G).

The iso20q variant interrupts germ layer differentiation

Our RPE differentiation method is spontaneous, based on hPSCs' propensity to generate a proportion of pigmented foci over time (Vugler et al., 2008). We hypothesized that the interruption of differentiation we observed is generalized to the germ layers. First, we monitored the early RPE

(B) Quantification of foci areas at day 16 of RPE differentiation in M7 WT (A1, A2) versus iso20q (A5, A9) variants. Data are presented as mean + SEM, and statistical significance was determined using Student's t test, two-tailed, n = 6, 3 independent experiments for each clonal line.

(C) Phase images of iso20q morphology at 10 days of RPE differentiation. Scale bar, 400 μm.

(D) Quantification of viability in the A2 WT versus A5, A9 iso20q clones at day 11 of RPE differentiation. Representative images illustrate cell death at day 16. Scale bar, 400 μm.

(E) Quantification of viability in the M8 and M13 WT/iso20q clonal pairs at day 11 of RPE differentiation and corresponding representative images. Scale bar, 400 μm.

(F) Flow cytometry analysis of caspase-3 in iso20q and WT variants at day 11 of RPE differentiation (image represents one independent experiment for each cell line).

(G) Overview of the iso20q phenotype during a spontaneous RPE differentiation.

*The data shown in (D) and (E) are presented as median of individual values of three replicates, and statistical significance was determined using Student's t test, two-tailed. See also Figure S2.

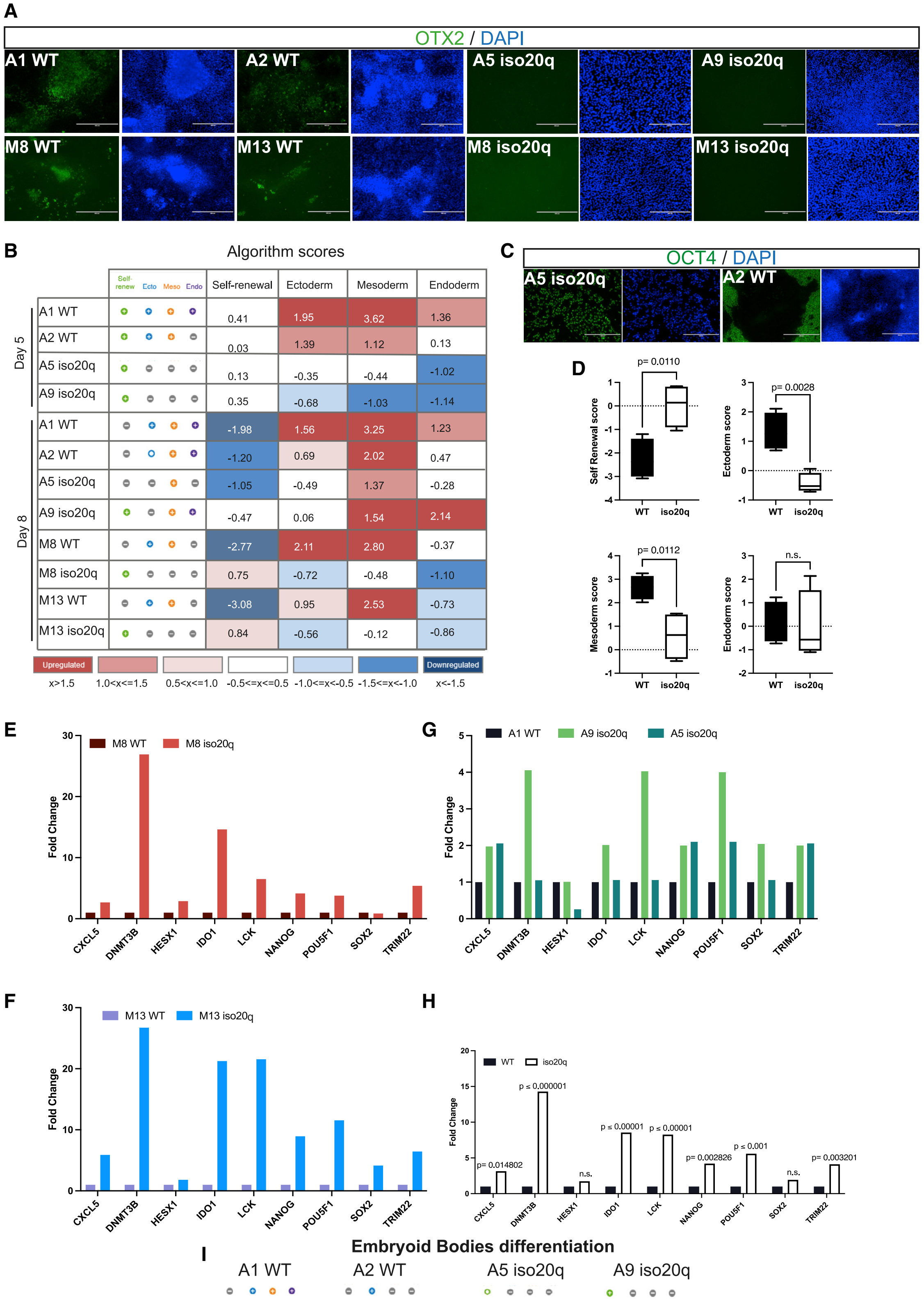

	Self-renewal	Ectoderm	Mesoderm	Endoderm
Day 5 A1 WT	0.41	1.95	3.62	1.36
Day 5 A2 WT	0.03	1.39	1.12	0.13
Day 5 A5 iso20q	0.13	-0.35	-0.44	-1.02
Day 5 A9 iso20q	0.35	-0.68	-1.03	-1.14
Day 8 A1 WT	-1.98	1.56	3.25	1.23
Day 8 A2 WT	-1.20	0.69	2.02	0.47
Day 8 A5 iso20q	-1.05	-0.49	1.37	-0.28
Day 8 A9 iso20q	-0.47	0.06	1.54	2.14
Day 8 M8 WT	-2.77	2.11	2.80	-0.37
Day 8 M8 iso20q	0.75	-0.72	-0.48	-1.10
Day 8 M13 WT	-3.08	0.95	2.53	-0.73
Day 8 M13 iso20q	0.84	-0.56	-0.12	-0.86

Upregulated						Downregulated
x>1.5	1.0<x<=1.5	0.5<x<=1.0	-0.5<=x<=0.5	-1.0<=x<-0.5	-1.5<=x<-1.0	x<-1.5

(legend on next page)

differentiation marker OTX2 by immunofluorescence at day 8 of the differentiation, prior to the onset of cell death in iso20q. We found that although OTX2 was expressed by WT lines, it was undetectable in iso20q variants, showing a failure in RPE lineage commitment (Figure 3A). Next, to assess whether the iso20q interrupts general differentiation, we performed the standardized pluripotency and three-germ-layer qPCR scorecard array on differentiated isogenic lines. Algorithm scores indicated that after 5 days of spontaneous differentiation the iso20q remained undifferentiated compared with WT and that none of the three germ layers was induced (Figures 3B, S3A, and S3B). As expected, by day 8, the WT clones progressed to fully exit the undifferentiated state, reflected in a negative score for self-renewing genes and positive score for the three germ layers (Figures 3B and S3C–S3E). At this later time point, the M7 A9 iso20q clone scored positive for self-renewing, mesoderm and endoderm genes while the M7 A5 clone lost the self-renewal score but upregulated mesoderm (Figures 3B, S3F, S3G, and S3D). However, immunofluorescence showed that the A5 clone actually retained homogeneous OCT4 at the protein levels up to day 8 in comparison with WT (Figure 3C). Knowing the ultimate fate of all the iso20q cells is apoptosis, the upregulation of genetic markers by M7 clones at day 8 is likely not biologically reflective of lineage commitment. Importantly, in the other two genetically distinct lines, M8 and M13, the scores of iso20q cells remained positive for self-renewal and negative for germ layers even after 8 days of differentiation (Figures 3B, S3H, S3I, and S3B). Moreover, by comparing all the iso20q lines with WT at day 8 we found that the self-renewal scores were significantly downregulated in WT but not in iso20q while ectoderm and mesoderm were significantly upregulated in WT and endoderm was unchanged (Figure 3D).

Score data revealed that the iso20q abnormality induces cells to retain genes associated with the undifferentiated status during differentiation. Therefore, we compared the gene expression levels of the self-renewal panel between the iso20q lines and their own WT counterparts, thus not against the standard reference. This analysis revealed that most self-renewal markers were overexpressed in the iso20q versus WT cells at day 8 (Figures 3E–3H). These results show that in iso20q variants, the core pluripotency network remains active despite a shift to spontaneous differentiating conditions.

Finally, we asked whether the iso20q germ layer blockage occurs also if hPSCs are placed under three-dimensional (3D) spontaneous differentiating conditions. Therefore, we validated the iso20q phenotype by performing scorecard analysis on embryoid bodies differentiation, confirming that the iso20q interruption of normal differentiation is generalized and not exclusive to a two-dimensional (2D) differentiation or the RPE lineage (Figures 3I, S4A, and S4B). Taken together, our phenotyping and gene expression data demonstrate that the iso20q variant interrupts hESCs spontaneous differentiation to the three germ layers in both 2D and 3D differentiating conditions.

DNMT3B-overexpressing iso20q variants are not nullipotent

Among the self-renewal genes that remained significantly upregulated in the iso20q variants we noted the chromosome 20q-localized gene *DNMT3B*, encoding for the DNA-methyltransferase 3 beta. Genome-wide *de novo* methylations by DNMT3B is indispensable during mammalian

Figure 3. The iso20q variant interrupts germ layer differentiation

(A) Immunofluorescence of WT and iso20q pairs stained for early RPE differentiation marker OTX2 at day 8 of spontaneous differentiation. (representative image of three independent experiments). Scale bar, 400 μm.

(B) Pluripotency scorecard algorithm results for WT and iso20q clonal pairs at day 5 (M7) and day 8 (all cell lines) of RPE differentiation. Scores for self-renewal and the three germ layers are calculated by comparison with global hPSCs reference standards.

(C) Immunofluorescence of WT A1 and iso20q A5 clonal pairs stained for OCT4 at day 8 of spontaneous differentiation (representative image of three independent experiments). Scale bar, 400 μm.

(D) Combined scorecard algorithm for self-renewal, ectoderm, mesoderm and endoderm for all WT and iso20q lines at day 8 of RPE differentiation. Data are presented as median plus min and max and statistical significance was determined using Student's t test, two-tailed, n = 4 cell lines for WT (M8 WT, M13 WT, M7 A1, M7 A2) and for iso20q (M8 iso20q, M13 iso20q, M7 A9, M7 A5). n.s., not significant.

(E) Gene expression for pluripotency-associated genes in the scorecard self-renew panel showing fold change of iso20q versus own WT counterpart for the M8 clonal pairs at day 8 of the RPE differentiation.

(F) Gene expression for pluripotency-associated genes in the scorecard self-renew panel showing fold change of iso20q versus own WT counterpart for the M13 cell line at day 8 of the RPE differentiation.

(G) Gene expression for pluripotency-associated genes in the scorecard self-renew panel showing fold change of iso20q versus own WT counterpart for M7 clonal pairs at day 8 of the RPE differentiation.

(H) Combined gene expression for pluripotency-associated genes in the scorecard self-renew panel showing fold change of iso20q versus WT at day 8 of the RPE differentiation. Statistical significance was determined using Student's t test, two-tailed, n = 3 cell lines for WT (M8 WT, M13 WT, M7 A1) and for iso20q (M9 iso20q, M13 iso20q, M7 A9). n.s., not significant.

See also Figures S3 and S4.

development as it establishes lineage-specific patterns after exit from pluripotency (Smith et al., 2012). DNMT3B is highly expressed in hPSCs as well as nullipotent embryonal carcinoma (EC) cells. Although *DNMT3B*-null hPSCs are viable, inhibition of DNMT3B catalytic activity induces apoptosis and differentiation of hPSCs but fail at differentiating nullipotent EC cells (Wongtrakoongate et al., 2014). DNMT3B deficiency accelerates neural and neural crest specification (Martins-Taylor et al., 2012). However, little is known of the effects of DNMT3B overexpression in hPSCs. We presupposed that *DNMT3B* would be overexpressed at the basal level in self-renewing iso20q hPSCs variants to reflect the trisomy of this chromosomal abnormality. Gene expression confirmed that, compared with WT cells, *DNMT3B* is upregulated in iso20q cells under routine self-renewing conditions alongside BCL-XL, also on the 20q arm (Figure 4A). Even in the one iso20q clone (M7 A5) in which *DNMT3B* was not upregulated by scorecard analysis at day 8, on a time course of differentiation it remains higher compared with WT cells (Figure S5A). Multi-omics profiling and whole-genome sequencing of the variants would be necessary for detecting variations in methylation imprinting caused by DNMT3B overexpression in iso20q.

Because iso20q cells are refractory to differentiation, we tested whether they respond to inhibition of DNMT3B like normal hPSCs or like nullipotent EC cells. We inhibited DNMT3B catalytic activity with the selective compound nanaomycin A (Kuck et al., 2010) for the first 3 days of spontaneous differentiation in the iso20q (M7 A9) and WT (M7 A2) isogenic lines (Figure 4B). After 8 days, we found that nanaomycin A had induced significant cell death (i.e., reduced viability) and signs of neuralization in WT cells (Figures 4C and 4D), in accord with previous reports (Martins-Taylor et al., 2012; Wongtrakoongate et al., 2014). In iso20q cells, we also observed low viability after nanaomycin A, but at percentages not different from its DMSO control, which remains significantly lower than the WT DMSO (Figures 4C and 4D). Therefore, inhibiting DNMT3B did not increase nor rescue the iso20q apoptotic phenotype. With respect to differentiation, we observed clear morphological changes in iso20q cells treated with nanaomycin A with the appearance of specialized-like cells and clumps in place of the typically undifferentiated cells of the variant (Figure 4C). Although this differentiation was not comparable with normal WT cells, it shows that iso20q cells have a capacity for specialization and are not nullipotent.

To elucidate the type of differentiation promoted by nanaomycin A, and in view of reports of DNMT3B inhibition stimulating neural crest and neural differentiation (Martins-Taylor et al., 2012), we compared the gene expression levels of the neurectoderm marker *PAX6* as well as the early neural, and RPE, marker *OTX2* in WT versus iso20q lines. We confirmed that nanaomycin A induced neuralization in WT cells via upregulation of *PAX6*, although *OTX2* was unchanged (Figure 4E). However, *PAX6* and *OTX2* remained significantly downregulated in iso20q compared with WT cells even after nanaomycin A (Figure 4E). These results show that inhibition of DNMT3B with nanaomycin A in iso20q was not sufficient to rescue spontaneous differentiation but neither induced neuralization. Indeed, treatment with nanaomycin A did not induce a significant downregulation of pluripotency-associated gene *NANOG* in iso20q cells (Figure 4F). Next, we hypothesized that iso20q variants might upregulate other germ layers rather than ectoderm after nanaomycin A, even if exit from pluripotency was not completed. Thus, we looked at gene expression levels of key transcriptional regulators of endoderm (*SOX17*), mesoderm (T), and trophoblast (*CDX2*) (Figures 4F–4I). To our surprise, we found that iso20q cells only significantly upregulated *CDX2* (Figure 4I), a phenomenon not seen in WT counterparts (Figure S5B), indicating a bias toward extra-embryonic lineages.

Our inhibition approach to DNMT3B catalytic activity has highlighted yet another difference in behavior between iso20q and WT cells, in particular the upregulation of extra-embryonic lineage markers. Further studies on selective knockout lines would be required to specifically test the involvement of the extra copy of DNMT3B in the iso20q phenotype.

Combined, our results show that (1) DNMT3B inhibition induces apoptosis and neural differentiation in WT but not iso20q cells; (2) iso20q cells are not nullipotent, as they are capable to respond with differentiation, skewed to extra-embryonic lineages, after DNMT3B inhibition; and (3) neither cell death nor spontaneous differentiation was rescued up to normal levels by inhibiting DNMT3B.

The iso20q variant retain differentiation competency toward extra-embryonic amnion

The iso20q is a multigenic and large abnormality spanning both copy number losses and gains of vast chromosomal regions. It is plausible that the iso20q phenotype results from a dose-dependent balance caused by the acquisitions of extra genetic copies on the 20q arm, losses of genes on the 20p arm or a combination of both. We rationalized that functional analyses centered on protein activity would elucidate the mechanisms driving the behavioral fitness of the variant. Consequently, we challenged the iso20q variants with a screening approach with the aim of finding factors capable of inducing their differentiation in our spontaneous system. We selected key pathways known to regulate human pluripotency and RPE differentiation. As our spontaneous RPE differentiation protocol is based

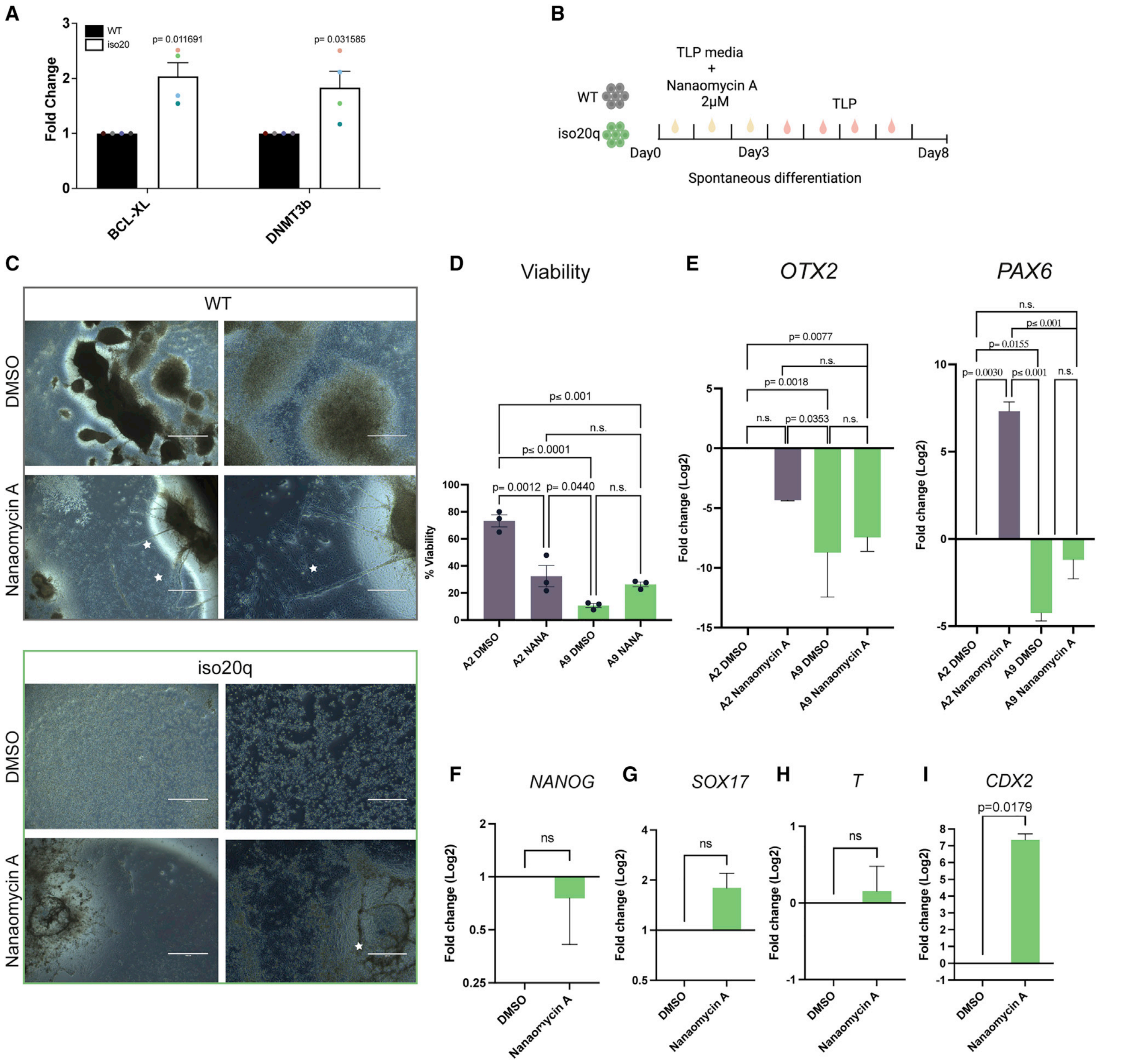

Figure 4. DNMT3B-overexpressing iso20q variants are not nullipotent

(A) Gene expression fold change for *BCL-XL* and *DNMT3B* in iso20q versus WT undifferentiated hESCs lines. Statistical significance was determined using Student's t test, two-tailed, n = 4 cell lines.

(B) Treatment regime of DNMT3B inhibition. DMSO or nanaomycin A (2 μM) were added for the first three days of differentiation in TLP media.

(C) Phase images of iso20q A9 and WT A2 lines at day 8 of differentiation following treatment with DMSO or nanaomycin A as shown in (B). White asterisks indicate differentiation. Scale bars, 1,000 and 400 μm.

(D) Percentage of viable cells at day 8 following treatment with DMSO or nanaomycin A as shown in (B). Data are presented as mean +/- SEM. Statistical significance was determined using one-way ANOVA, multiple comparisons, n = 3. n.s., not significant.

(E) Gene expression of ectodermal markers *OTX2* and *PAX6* in iso20q A9 relative to WT A2 at day 8 of differentiation following treatment with DMSO or nanaomycin A as shown in (B). Data are represented as mean + SEM, and statistical significance was determined using one-way ANOVA, multiple comparisons, n = 3. n.s., not significant.

(F–I) Gene expression of pluripotency-associated marker (F) *NANOG*, (G) endoderm marker *SOX17*, (H) mesoderm marker *T*, and (I) trophectoderm marker *CDX2* in iso20q A9 at day 8 of differentiation following treatment with DMSO or nanaomycin A as shown in (B). Data are presented as mean + SEM, and statistical significance was determined using Student's t test, two-tailed, n = 3. ns, not significant. See also Figure S5.

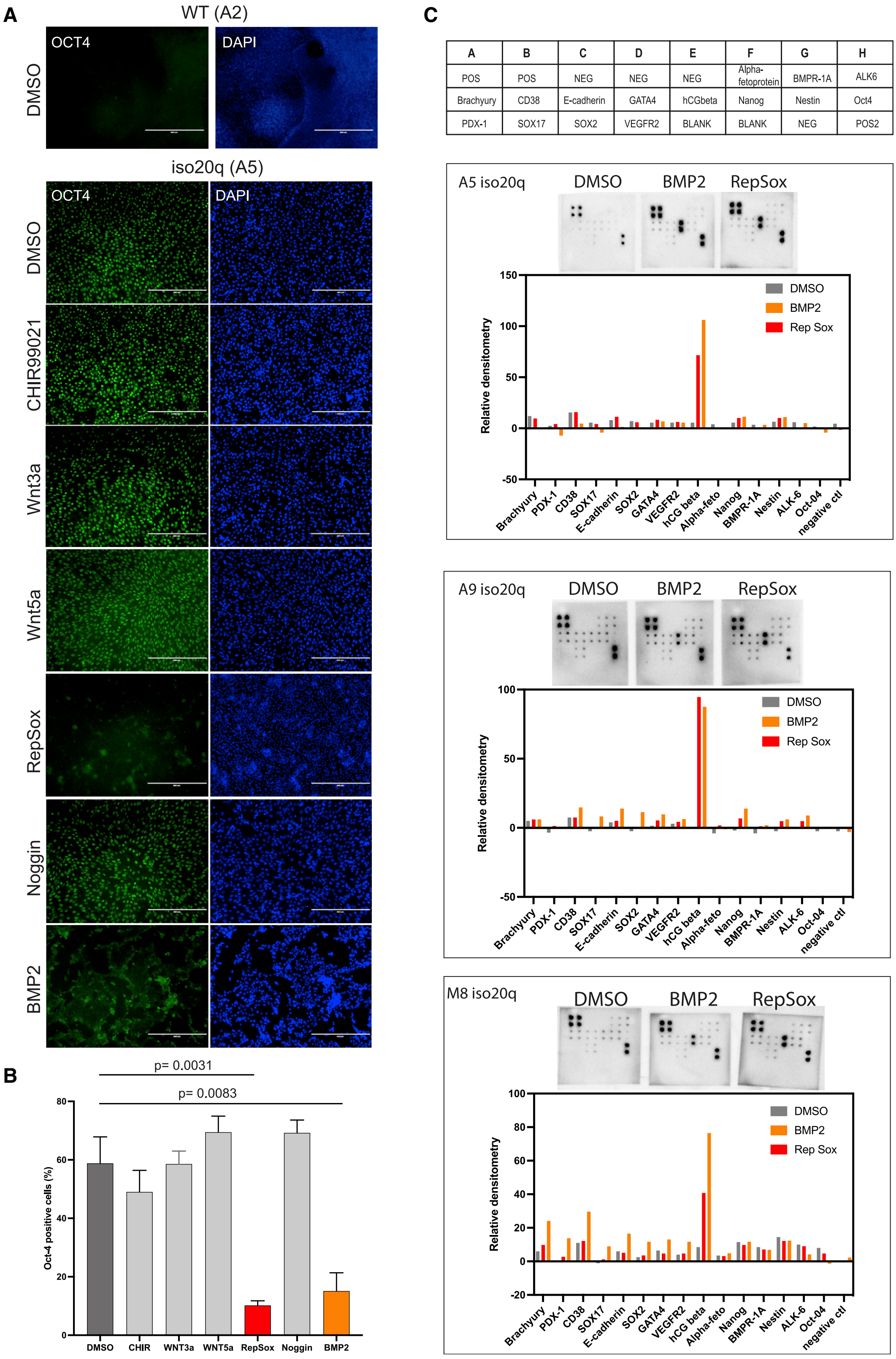

A	B	C	D	E	F	G	H
POS	POS	NEG	NEG	NEG	Alpha-fetoprotein	BMPR-1A	ALK6
Brachyury	CD38	E-cadherin	GATA4	hCGbeta	Nanog	Nestin	Oct4
PDX-1	SOX17	SOX2	VEGFR2	BLANK	BLANK	NEG	POS2

(legend on next page)

on FGF2 withdrawal, we did not modulate its signaling. We supplemented the culture with BMP2, whose signaling is crucial for hPSC differentiation and RPE biology (Gordeeva, 2019; O'Hara-Wright and Gonzalez-Cordero, 2020; Pera and Tam, 2010). Notably, BMP2 is monosomic in iso20q variants. We inhibited TGF-β receptor ALK5 with the RepSox compound, which plays a master role in maintenance of pluripotency and RPE differentiation (Gordeeva, 2019; Pauklin and Vallier, 2015; Radeke et al., 2015). Furthermore, we used Noggin to antagonize BMP signaling, as it has a role in both pluripotency and eye development (Gordeeva, 2019; O'Hara-Wright and Gonzalez-Cordero, 2020; Pera and Tam, 2010). Finally, we activated Wnt signaling, important in both retina development and pluripotency (Gordeeva, 2019; Leach et al., 2015; O'Hara-Wright and Gonzalez-Cordero, 2020; Steinfeld et al., 2013), with both Wnt3a or Wnt5a, as well as CHIR99021, a widely used GSK3 inhibitor and Wnt pathway activator (Leach et al., 2015). We treated our lead iso20q clonal pairs (A5 and A9) with the above panel of factors for 8 days of spontaneous differentiation and used OCT4 protein expression as primary readout of pluripotency. We considered that by day 8, any significant change to the iso20q differentiation block should be detectable with an immune screening for a key marker associated with pluripotent cells. Indeed, whereas the WT control lost almost all OCT4 by day 8, the iso20q cells did not show signs of differentiation and remained OCT4 positive (Figures 5A and S5C). OCT4 remained unchanged after treatment with CHIR99021, Wnt3a, Wnt5a, and Noggin (Figures 5A and S5C). We also noted that treatment with CHIR99021 seemed to accelerate the cell death of the iso20q (data not shown). Conversely, inhibition of TGF-β with RepSox or addition of BMP2 both led to a morphological difference in iso20q clones with many OCT4-negative cell aggregates, pointing to a shift out of the differentiation block (Figures 5A and S5C). In particular, cells treated with RepSox and BMP2 showed aggregates of cells with an epithelial morphology with no signs of the flattened and apoptotic cells as seen in the iso20q phenotype (Figure S5E). We quantified this effect by measuring OCT4-positive cells in all conditions and found that only RepSox and BMP2 significantly reduced its expression (Figures 5B and S5D). The similar outcomes of these two treatments can be explained by the close interplay between TGF-β and BMP signaling (Gordeeva, 2019; Pauklin and Vallier, 2015), whereby inhibition of TGF-β effectively shifts the balance toward pro-differentiative BMP effectors. Next, we investigated the nature of the cell types produced after treatments with RepSox and BMP2 on a multiplex protein array for key pluripotency markers and germ layers. Qualitative and semi-quantitative analysis of the arrays showed that all three iso20q lines treated with RepSox or BMP2 equally upregulated the human chorionic gonadotropin beta (hCGbeta) protein above all other markers (Figure 5C). These data demonstrate that BMP2 and RepSox have pushed iso20q out of the differentiation block by promoting differentiation to cells expressing high levels of hCGbeta.

HCG beta is typically expressed by the trophectoderm and trophoblast cells, but it can also be secreted by somatic cells and tumors (Lee et al., 2016). During mammalian embryogenesis, the first lineage segregation that occurs is between the epiblast and the trophectoderm, the outer layer of the blastocyst giving rise to the extra-embryonic tissues of the placenta (Guo et al., 2021). Researchers have reported hPSCs acquiring a trophoblast identity in response to BMP4, which binds type1 receptors similarly to BMP2 (Dong et al., 2020; Gamage et al., 2016). However, conventional human PSCs represent cells at the post-implantation stage while developmental plasticity for trophoblast is maintained until implantation (Guo et al., 2021). It has been clarified that hPSCs, contrary to naive cells, have a restricted potential for the post-implantation extra-embryonic lineage, the amnion, which however shares most key markers in common with the trophectoderm (Guo et al., 2021). Consequently, we investigated whether our BMP2 and RepSox-treated iso20q hPSCs variants have acquired an amnion-like fate by looking at a panel of markers previously used to characterize placental cells and hPSC-derived trophoblast and amnion (Dong et al., 2020; Gamage et al., 2016; Guo et al., 2021; Lee et al., 2016). Gene expression showed that iso20q variants differentiated as a result of BMP2 had upregulated levels of *hCGb*, *KRT7*, *GATA3*, and *CDX2* after 8 days, while RepSox upregulated all markers but *CDX2* (Figure 6A).

Figure 5. BMP2 and TGF-β inhibition induce differentiation of iso20q cells

(A) Representative immunofluorescence images of iso20q A5 clones treated with DMSO or CHIR99021 (1 μM), Wnt3a (100 ng/mL), Wnt5a (100 ng/mL), RepSox (100nM), Noggin (200 ng/mL), or BMP2 (100 ng/mL) for 8 days of spontaneous RPE differentiation and stained with OCT4 and DAPI. The WT A2 clone treated with DMSO is shown as control. Scale bars, 400 μm.

(B) Quantification of OCT4-positive cells in A5 iso20q clones treated as shown in (A). Data are presented as mean + SEM, and statistical significance was determined using Student's t test, one-tailed; n = 3 independent experiments.

(C) Human stem cell proteomic array blots and corresponding relative densitometry for iso20q clones (A5, A9, and M8) treated with DMSO control, 100 ng/mL BMP2, or 100nM RepSox for 8 days of RPE differentiation. Marker localization shown in array map.

See also Figure S5.

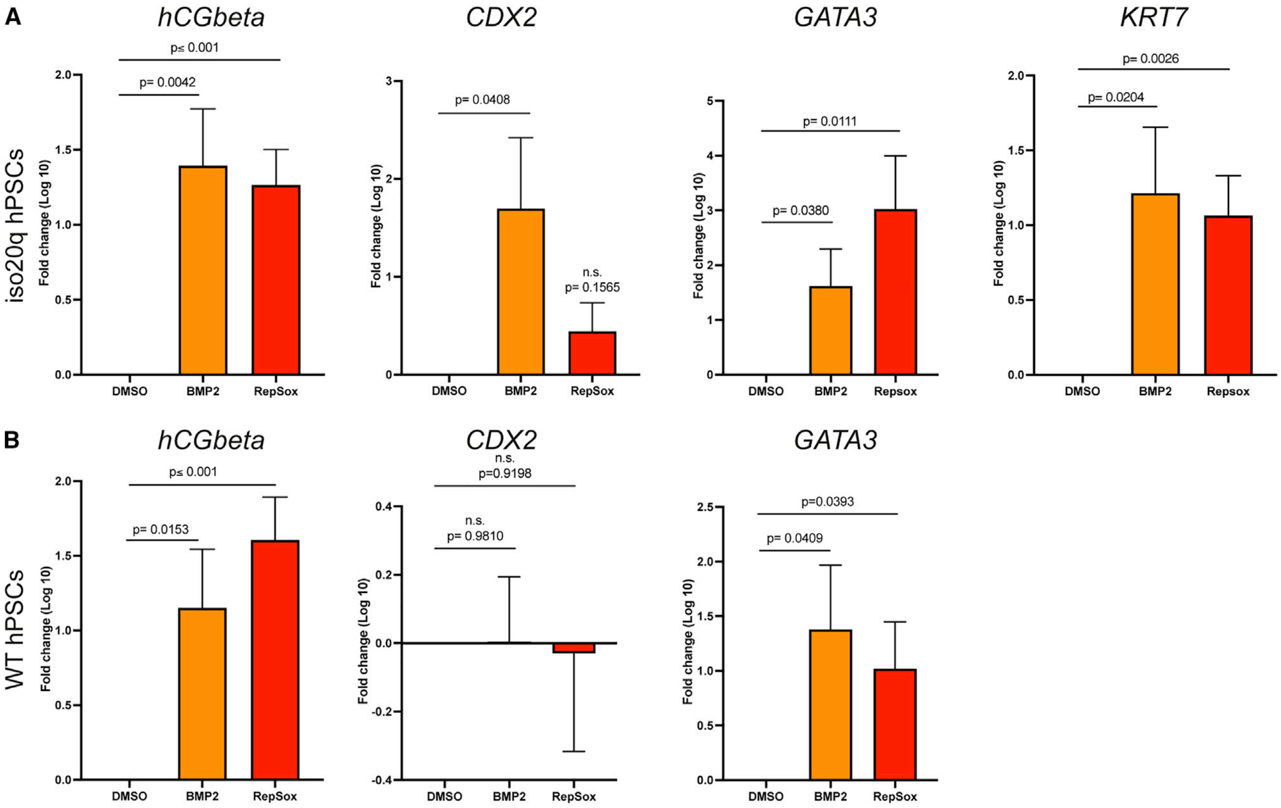

Figure 6. The iso20q variant retain differentiation competency toward extra-embryonic amnion

(A) Gene expression of trophoblast markers in iso20q A5 and A9 clones treated with 100 ng/mL BMP2 or 100nM RepSox for 8 days of the RPE differentiation. Data are presented as mean + SEM, and statistical significance was determined using Student's t test, one-tailed; n = 6 (3 independent experiments for each clonal line). n.s., not significant.

(B) Gene expression of trophoblast markers in WT A1 and A2 clones treated with 100 ng/mL BMP2 or 100nM RepSox for 8 days of the RPE differentiation. Data are presented as mean + SEM, and statistical significance was determined using Student's t test, one-tailed; n = 6 (3 independent experiments for each clonal line). n.s., not significant.

See also Figure S6.

However, normal WT cells treated with the same conditions also upregulated *hCGb* and *GATA3* but not *CDX2* and *KRT7*, which was even below detectable levels (Figure 6B). The upregulation of these markers in normal clones show that our rescue treatment had unintentionally created the conditions of trophoblast-like differentiation of primed hESCs. Differences in marker expression between iso20q and wild-type clones, especially the absence of *KRT7* (Dong et al., 2020), suggest that hESCs carrying the iso20q abnormality might diverge during trophoblast/amnion differentiation forming specific subtypes. Indeed, similar results were seen with M8 isogenic pairs (Figure S6A). Moreover, M13 iso20q showed the greatest bias toward trophectoderm as their WT cells did not significantly upregulate any extra-embryonic markers (Figure S6B). These results show that iso20q variants are biased for amnion-like differentiation signals and undertake their own trajectory within this lineage.

Directed differentiation protocols mask iso20q underlying developmental defect

The differentiation protocol used throughout this work is spontaneous. To understand the effect of iso20q in the context of directed differentiation, we performed directed trilineage differentiation with standardized protocols on all isogenic lines (Figure S7). We found that iso20q cells were capable to express key differentiation markers for endoderm, mesoderm, and ectoderm (Figure S7). These results show that iso20q cells can be pushed toward a specific lineage with enough cues to begin and progress differentiation but fail under spontaneous or embryoid body differentiation. Therefore, the stark iso20q variants' developmental defects we discovered under a spontaneous system are masked by common directed differentiation methods. Combined, our findings back a model in which genetically abnormal iso20q hPSCs lack spontaneous developmental competency for epiblast

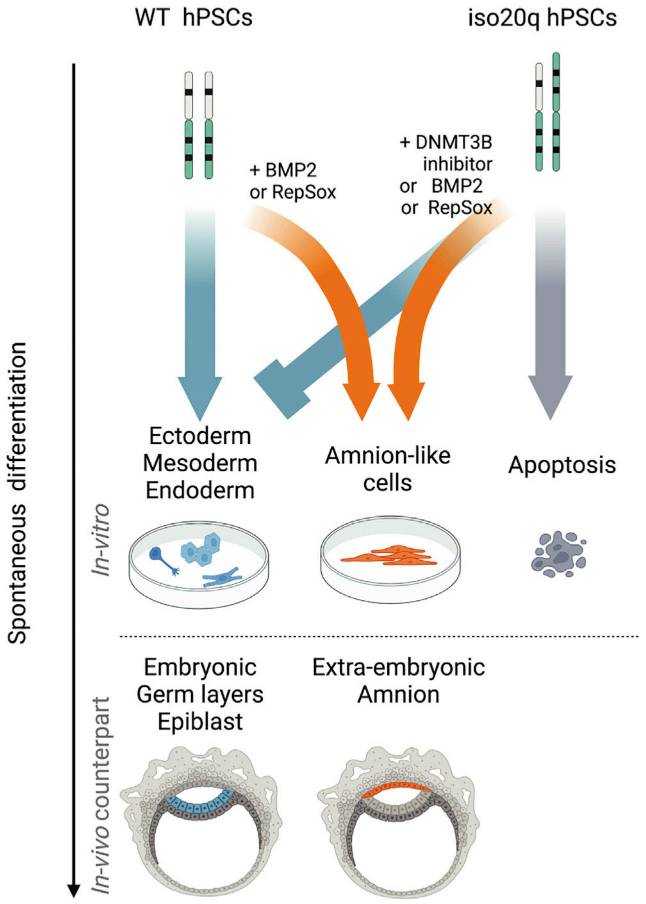

Figure 7. The iso20q genotype-phenotype relationship
Diagram illustrating the phenotype of hPSCs carrying the iso20q abnormality in comparison with WT cells during spontaneous differentiation.

germ layers while display a bias for extra-embryonic lineages (Figure 7).

DISCUSSION

Understanding the role of recurrent chromosomal abnormalities of human pluripotent stem cells during differentiation is a key objective for the safe and effective use of these cells in research and clinic. Our findings reveal that acquisition of the iso20q abnormality disrupts hPSCs lineage segregation. Indeed, when placed under spontaneous differentiation conditions, hPSCs carrying the iso20q remain in an undifferentiated "limbo" until they are depleted by apoptosis. Moreover, when *de novo* DNMT3B methylation is inhibited or amnion is induced, iso20q cells upregulate more extra-embryonic markers compared with WT. Thus, abnormal iso20q cells are biased to progress toward an extra-embryonic but not an epiblast developmental trajectory. The seemingly clear-cut iso20q phenotype we describe in this study has several implications that interest interdisciplinary areas of basic and translational research using pluripotent stem cells.

A first important implication relates to the field of regenerative medicine. The primary aim of this study was to evaluate the effect of iso20q during an RPE differentiation protocol currently in clinical trials (da Cruz et al., 2018). Reassuringly, our results demonstrate that the iso20q variant self-eliminate via apoptosis after only two weeks of RPE differentiation, therefore any risk for contamination or tumorigenicity for such cell therapy product is likely eradicated. However, the impact of iso20q on differentiation could be masked to researchers using chemically-directed methods, especially those using BMP2 and TGF-β inhibition. Although we cannot predict the effect of iso20q variants in each differentiation protocol, we can assume that it would affect pluripotent stem cells differentiation capacity on a fundamental level. Even if iso20q variants were, under the right conditions, led to differentiate in a seemingly normal fashion, their genomic imbalance questions the quality and safety of their derivatives. An effective strategy would be to routinely test cell lines for common abnormalities and produce large cell banks of normal clones. However, it is not necessarily true that an abnormality poses a safety risk and therefore those needs to be assessed on a case-by-case basis for each differentiation protocol, especially those developed for cell therapy.

A second implication of our results is that the iso20q is to date the first known example of a single defined genetic aberration that interrupts the spontaneous differentiation of hPSCs. Acquisition of selective advantage of mutated lines over wild-type cells has been reported primarily as a result of suppression of apoptosis (Avery et al., 2013), faster cell division (Ben-David et al., 2014), inhibition of wild-type cell growth(Lee et al., 2015), reduced or delayed differentiation (Ben-David et al., 2014; Markouli et al., 2019; Werbowetski-Ogilvie et al., 2009), but not because of interruption of germ layer differentiation. Compared with reports of reduced differentiation potential of hPSCs with other common abnormalities, our study shows that the presence of an iso20q impairs general, undirected, three-germ-layer differentiation while showing a bias toward extra-embryonic lineages. Interestingly, although iso20q cells fail to differentiate spontaneously, when pushed by targeted directed differentiation can form cell types of the three lineages. This suggests that iso20q impairment is revealed under spontaneous systems but is masked by commonly used directed methods, which are able to overcome its developmental shortcomings. In light of these results, it would be important to test other abnormalities under spontaneous protocols, like ours, to establish their basic level of differentiation potential and make better comparisons with iso20q.

Our findings open the key question of whether the resistance to spontaneous differentiation is also a cause rather than an effect of the selective advantage for iso20q. Such chain of causality could be entangled by cell competition experiments within a pluripotent niche, similar to those we recently performed (Price et al., 2021).

Third, our studies highlight an undepreciated role of chromosome 20 in pluripotency. Chromosome 20 is already a big actor in the field because of the 20q11.21 copy number variant being the most frequent aberration in hPSC cultures, as the antiapoptotic *BCL2L1* gene, localized in the amplicon, is driving the growth advantage (Avery et al., 2013). Our findings show that dose-dependent dysregulation of genes on chromosome 20 can alter pluripotency, namely, the ability to differentiate into the three germ layers. This loss of differentiation capacity happens in parallel with iso20q variants inability to downregulate core pluripotency-associated genes. Importantly, iso20q hPSCs respond to differentiation by stopping self-renewal and undergoing apoptosis, suggesting the presence of a quality check-point. We propose that the iso20q abnormality is intercepted by developmental mechanisms that prevent highly mutated cells to form embryonic, but as we have seen here, not extra-embryonic tissue. Inhibition of DNMT3B in variant cells upregulated *CDX2*, indicating that iso20q cells have a bias for the extra-embryonic lineage independently of amnion-inducing BMP. Given the multigenic and dose-dependent nature of this aberration, multi-omics approaches would be required to reveal the far-reaching signature of the iso20q on pluripotent stem cells biology and fitness.

Finally, our findings have broader implications for trophoblast research. The iso20q abnormality is not an artifact emerging during selective pressure in stem cell cultures but is found in human amniocentesis (Receveur et al., 2017). According to clinical reports, the iso20q is a very rare and mosaic abnormality with around 30 pre-natal cases recorded of which only five with fetal malformations (Receveur et al., 2017). In many cases the karyotype on fetal tissue is normal even if iso20q is detected at amniocentesis. The placenta has recently emerged as a genetically heterogeneous organ where abnormal and mutated cells of fetal origin drift, with the bottleneck occurring as early as the zygote (Coorens et al., 2021). This means that developmental control mechanisms segregate abnormal cells from the epiblast germ layers to the more permissive extra-embryonic tissues. Indeed, it has been reported in mouse models, and later in humans, that the embryo has an intrinsic ability to select against abnormal aneuploid cells (Bolton et al., 2016; Yang et al., 2021). Therefore, it would be interesting to investigate the presence of iso20q in phylogenetic studies of human placenta tissues. Considering both our *in vitro* phenotype and the clinical records, it is conceivable that iso20q cells are removed at the level of the epiblast via apoptosis or are segregated to the extra-embryonic space to protect the genomic integrity of the embryo. In support of this model, studies of gastrulation in embryonic stem cell demonstrated that heterogeneously aneuploid cells are depleted from the epiblast but not from the trophectoderm in response to BMP4 (Yang et al., 2021). However, our iso20q isogenic study reveal that aneuploidy depletion can occur independently to BMP. Indeed, apoptosis is the default fate of iso20q cells after spontaneous differentiation and does not correlate with BMP2-induced amnion differentiation. Furthermore, we observed that iso20q cells were depleted during spontaneous but not directed differentiation. This suggests that compensation mechanisms could still direct iso20q toward normal development; whether this occurs physiologically for iso20q in the embryo remains unknown. Therefore, spontaneous 3D models of early development would be most appropriate to investigate iso20q and to gather insight into aneuploidy depletion mechanisms.

Overall, we discovered a genotype-phenotype correlation for the iso20q abnormality, which represents an *in vitro* example of aneuploidy depletion from embryonic tissue in accord with its own clinical records. Our iso20q isogenic lines offer a karyotypically defined platform to study cell segregation of genomic aberrations in models of early embryogenesis, such as blastoids and gastruloids (Yang et al., 2021; Yu et al., 2021), and to gain mechanistic insight into such important yet elusive processes of human development.

EXPERIMENTAL PROCEDURES

Resource availability

Corresponding author

Further information and requests for resources and reagents should be directed to and will be fulfilled by the corresponding author, Loriana Vitillo (l.vitillo@ucl.ac.uk).

Materials availability

Human embryonic stem cell lines used in this study are available from the UK Stem Cell Bank. Isogenic lines are available upon reasonable request.

Cell lines

Derivation of the MasterShef-7, MasterShef-8, and MasterShef-13 cell lines was performed in the Stem Cell Derivation Facility at the Centre for Stem Cell Biology, University of Sheffield, under HFEA license R115-8-A (Centre 0191) and HTA license 22510, in a clean-room setting, following strict standard operating procedures. All cell lines have been deposited at the UK Stem Cell bank.

Karyotyping and FISH

Karyotyping and FISH were performed by Sheffield Diagnostic Genetic Services (Sheffield Children's Hospital). For karyotyping, cells were arrested in metaphase with KaryoMAX Colcemid (Thermo

Fisher Scientific) followed by a methanol-based fixative method as previously described (Laing et al., 2019). FISH analysis was executed using the D20S108 (20q12), RP5-857M17(BCL2L1) and 20p telomere probes on 100 interphase cells fixed as previously described (Laing et al., 2019).

Cell culture

Undifferentiated hESCs were cultured on Nutristem hPSC XF medium (Biological Industries) on Laminin 521 (Biolamina) coated wells. hESCs were passaged with 0.5 mM EDTA (Invitrogen) every 5 days and frozen in Bambanker (BB03; Anachem).

hESC-RPE differentiation

hESCs were passaged with EDTA and re-plated onto 6-well plates coated with Matrix Matrigel hESc qualified (734-1440; Corning) in Nutristem medium. Colonies were grown until confluence with daily media change. Differentiation to RPE was initiated by transitioning cultured to differentiation media TLP consisting of knockout DMEM, 20% knockout serum replacement, 1% non-essential amino acids, 1 mM L-glutamine, and 0.1 mM β-mercaptoethanol (Invitrogen, Life Technologies) as previously described (Carter et al., 2016; da Cruz et al., 2018). TLP was replaced every week day for the first 14 days of differentiation followed by a twice-weekly regime.

hPSC TaqMan scorecard assay

In all, 1 μg total RNA was retro-transcribed using the high-capacity cDNA Reverse transcription kit (Applied Biosystems). Samples were prepared for the assay in TaqMan Fast Advanced Mastermix (Applied Biosystems) and loaded onto either a 384- or 96-well TaqMan hPSC scorecard panel following manufacturer instruction (A15870, A15876; Applied Biosystems). Transcript levels were acquired using the Scorecard template on a 12K Quantstudio or StepOne Plus (Applied Biosystems) for 384- and 96-well formats, respectively. Transcript results were analyzed using the web-based hPSC scorecard software.

Statistical analysis and reproducibility

Statistical analysis was performed on a minimum of triplicate independent experiments in GraphPad Prism 9, with p values <0.05 defining statistical significance. Images are shown as the representative of all independent experiments.

SUPPLEMENTAL INFORMATION

Supplemental information can be found online at https://doi.org/10.1016/j.stemcr.2023.01.007.

AUTHOR CONTRIBUTIONS

L.V. designed, performed, and analyzed the experiments and wrote the manuscript. F.A. assisted with experiments and analysis. Z.H. supervised the derivation of the clones and wrote parts of the manuscript. D.S. performed flow cytometry and assisted with scorecard assays. O.L. performed 20q qPCR assay. D.B. performed karyology and FISH on the cell lines. I.B. and P.C. supervised the study. All authors commented and approved the manuscript.

ACKNOWLEDGEMENTS

This work was supported by the UK Regenerative Medicine Platform (grants MR/L012537/1 and MR/R015724/1) (Z.H., D.S., O.L., I.B.), the Lincy Foundation, The London Project to Cure Blindness: funding toward the Production of a Cell Therapy for Late Stage Age-Related Macular Degeneration – [P12761]; CIRM (California Institute of Regenerative Medicine) [LA1_C2-02086]; MRC, Stem Cell Based Treatment Strategy for Age-Related Macular Degeneration [G1000730] (L.V., F.A., P.C.). We thank Sian Gregory, Richard Weightman, and Allan Shaw for support with generating the clones. We thank Peter Andrews for discussion. Diagrams created with BioRender.com.

CONFLICT OF INTERESTS

Z.H. is a co-founder and majority shareholder of Regenerative Cell Therapy Consulting Limited. I.B. is a member of the Scientific Advisory Boards of WiCell and Broken String Biosciences.

Received: June 13, 2022
Revised: January 17, 2023
Accepted: January 18, 2023
Published: February 16, 2023

REFERENCES

Andrews, P.W., Ben-David, U., Benvenisty, N., Coffey, P., Eggan, K., Knowles, B.B., Nagy, A., Pera, M., Reubinoff, B., Rugg-Gunn, P.J., and Stacey, G.N. (2017). Assessing the safety of human pluripotent stem cells and their derivatives for clinical applications. Stem Cell Rep. *9*, 1–4. https://doi.org/10.1016/j.stemcr.2017.05.029.

Avery, S., Hirst, A.J., Baker, D., Lim, C.Y., Alagaratnam, S., Skotheim, R.I., Lothe, R.A., Pera, M.F., Colman, A., Robson, P., et al. (2013). BCL-XL mediates the strong selective advantage of a 20q11.21 amplification commonly found in human embryonic stem cell cultures. Stem Cell Rep. *1*, 379–386. https://doi.org/10.1016/j.stemcr.2013.10.005.

Baker, D., Hirst, A.J., Gokhale, P.J., Juarez, M.A., Williams, S., Wheeler, M., Bean, K., Allison, T.F., Moore, H.D., Andrews, P.W., and Barbaric, I. (2016). Detecting genetic mosaicism in cultures of human pluripotent stem cells. Stem Cell Rep. *7*, 998–1012. https://doi.org/10.1016/j.stemcr.2016.10.003.

Ben-David, U., Arad, G., Weissbein, U., Mandefro, B., Maimon, A., Golan-Lev, T., Narwani, K., Clark, A.T., Andrews, P.W., Benvenisty, N., and Carlos Biancotti, J. (2014). Aneuploidy induces profound changes in gene expression, proliferation and tumorigenicity of human pluripotent stem cells. Nat. Commun. *5*, 4825. https://doi.org/10.1038/ncomms5825.

Bolton, H., Graham, S.J.L., Van der Aa, N., Kumar, P., Theunis, K., Fernandez Gallardo, E., Voet, T., and Zernicka-Goetz, M. (2016). Mouse model of chromosome mosaicism reveals lineage-specific depletion of aneuploid cells and normal developmental potential. Nat. Commun. *7*, 11165. https://doi.org/10.1038/ncomms11165.

Carter, D.A., Smart, M.J.K., Letton, W.V.G., Ramsden, C.M., Nommiste, B., Chen, L.L., Fynes, K., Muthiah, M.N., Goh, P., Lane, A., et al. (2016). Mislocalisation of BEST1 in iPSC-derived retinal pigment epithelial cells from a family with autosomal dominant

vitreoretinochoroidopathy (ADVIRC). Sci. Rep. *6*, 33792. https://doi.org/10.1038/srep33792.

Coorens, T.H.H., Oliver, T.R.W., Sanghvi, R., Sovio, U., Cook, E., Vento-Tormo, R., Haniffa, M., Young, M.D., Rahbari, R., Sebire, N., et al. (2021). Inherent mosaicism and extensive mutation of human placentas. Nature *592*, 80–85. https://doi.org/10.1038/s41586-021-03345-1.

da Cruz, L., Fynes, K., Georgiadis, O., Kerby, J., Luo, Y.H., Ahmado, A., Vernon, A., Daniels, J.T., Nommiste, B., Hasan, S.M., et al. (2018). Phase 1 clinical study of an embryonic stem cell-derived retinal pigment epithelium patch in age-related macular degeneration. Nat. Biotechnol. *36*, 328–337. https://doi.org/10.1038/nbt.4114.

Dong, C., Beltcheva, M., Gontarz, P., Zhang, B., Popli, P., Fischer, L.A., Khan, S.A., Park, K.M., Yoon, E.J., Xing, X., et al. (2020). Derivation of trophoblast stem cells from naive human pluripotent stem cells. Elife *9*, e52504. https://doi.org/10.7554/eLife.52504.

Fazeli, A., Liew, C.G., Matin, M.M., Elliott, S., Jeanmeure, L.F.C., Wright, P.C., Moore, H., and Andrews, P.W. (2011). Altered patterns of differentiation in karyotypically abnormal human embryonic stem cells. Int. J. Dev. Biol. *55*, 175–180. https://doi.org/10.1387/ijdb.103177af.

Gamage, T.K., Chamley, L.W., and James, J.L. (2016). Stem cell insights into human trophoblast lineage differentiation. Hum. Reprod. Update *23*, 77–103. https://doi.org/10.1093/humupd/dmw026.

Garber, K. (2015). RIKEN suspends first clinical trial involving induced pluripotent stem cells. Nat. Biotechnol. *33*, 890–891. https://doi.org/10.1038/nbt0915-890.

Gordeeva, O. (2019). TGFbeta family signaling pathways in pluripotent and teratocarcinoma stem cells' fate decisions: balancing between self-renewal, differentiation, and cancer. Cells *8*, 1500. https://doi.org/10.3390/cells8121500.

Guo, G., Stirparo, G.G., Strawbridge, S.E., Spindlow, D., Yang, J., Clarke, J., Dattani, A., Yanagida, A., Li, M.A., Myers, S., et al. (2021). Human naive epiblast cells possess unrestricted lineage potential. Cell Stem Cell *28*, 1040–1056.e6. https://doi.org/10.1016/j.stem.2021.02.025.

Halliwell, J., Barbaric, I., and Andrews, P.W. (2020). Acquired genetic changes in human pluripotent stem cells: origins and consequences. Nat. Rev. Mol. Cell Biol. *21*, 715–728. https://doi.org/10.1038/s41580-020-00292-z.

International Stem Cell Initiative, Amps, K., Andrews, P.W., Anyfantis, G., Armstrong, L., Avery, S., Baharvand, H., Baker, J., Baker, D., Munoz, M.B., Beil, S., et al. (2011). Screening ethnically diverse human embryonic stem cells identifies a chromosome 20 minimal amplicon conferring growth advantage. Nat. Biotechnol. *29*, 1132–1144. https://doi.org/10.1038/nbt.2051.

Keller, A., Dziedzicka, D., Zambelli, F., Markouli, C., Sermon, K., Spits, C., and Geens, M. (2018). Genetic and epigenetic factors which modulate differentiation propensity in human pluripotent stem cells. Hum. Reprod. Update *24*, 162–175. https://doi.org/10.1093/humupd/dmx042.

Kobold, S., Guhr, A., Mah, N., Bultjer, N., Seltmann, S., Seiler Wulczyn, A.E.M., Stacey, G., Jie, H., Liu, W., Löser, P., and Kurtz, A. (2020). A manually curated database on clinical studies involving cell products derived from human pluripotent stem cells. Stem Cell Rep. *15*, 546–555. https://doi.org/10.1016/j.stemcr.2020.06.014.

Kuck, D., Caulfield, T., Lyko, F., and Medina-Franco, J.L. (2010). Nanaomycin A selectively inhibits DNMT3B and reactivates silenced tumor suppressor genes in human cancer cells. Mol. Cancer Ther. *9*, 3015–3023. https://doi.org/10.1158/1535-7163.MCT-10-0609.

Laing, O., Halliwell, J., and Barbaric, I. (2019). Rapid PCR assay for detecting common genetic variants arising in human pluripotent stem cell cultures. Curr. Protoc. Stem Cell Biol. *49*, e83. https://doi.org/10.1002/cpsc.83.

Leach, L.L., Buchholz, D.E., Nadar, V.P., Lowenstein, S.E., and Clegg, D.O. (2015). Canonical/beta-catenin Wnt pathway activation improves retinal pigmented epithelium derivation from human embryonic stem cells. Invest. Ophthalmol. Vis. Sci. *56*, 1002–1013. https://doi.org/10.1167/iovs.14-15835.

Lee, C.Q.E., Gardner, L., Turco, M., Zhao, N., Murray, M.J., Coleman, N., Rossant, J., Hemberger, M., and Moffett, A. (2016). What is trophoblast? A combination of criteria define human first-trimester trophoblast. Stem Cell Rep. *6*, 257–272. https://doi.org/10.1016/j.stemcr.2016.01.006.

Lee, C.T., Bendriem, R.M., Kindberg, A.A., Worden, L.T., Williams, M.P., Drgon, T., Mallon, B.S., Harvey, B.K., Richie, C.T., Hamilton, R.S., et al. (2015). Functional consequences of 17q21.31/WNT3-WNT9B amplification in hPSCs with respect to neural differentiation. Cell Rep. *10*, 616–632. https://doi.org/10.1016/j.celrep.2014.12.050.

Lund, R.J., Närvä, E., and Lahesmaa, R. (2012). Genetic and epigenetic stability of human pluripotent stem cells. Nat. Rev. Genet. *13*, 732–744. https://doi.org/10.1038/nrg3271.

Maeda, T., Sugita, S., Kurimoto, Y., and Takahashi, M. (2021). Trends of stem cell therapies in age-related macular degeneration. J. Clin. Med. *10*, 1785. https://doi.org/10.3390/jcm10081785.

Mandai, M., Watanabe, A., Kurimoto, Y., Hirami, Y., Morinaga, C., Daimon, T., Fujihara, M., Akimaru, H., Sakai, N., Shibata, Y., et al. (2017). Autologous induced stem-cell-derived retinal cells for macular degeneration. N. Engl. J. Med. *376*, 1038–1046. https://doi.org/10.1056/NEJMoa1608368.

Markouli, C., Couvreu De Deckersberg, E., Regin, M., Nguyen, H.T., Zambelli, F., Keller, A., Dziedzicka, D., De Kock, J., Tilleman, L., Van Nieuwerburgh, F., et al. (2019). Gain of 20q11.21 in human pluripotent stem cells impairs TGF-beta-dependent neuroectodermal commitment. Stem Cell Rep. *13*, 163–176. https://doi.org/10.1016/j.stemcr.2019.05.005.

Markouli, C., De Deckersberg, E.C., Dziedzicka, D., Regin, M., Franck, S., Keller, A., Gheldof, A., Geens, M., Sermon, K., and Spits, C. (2021). Sustained intrinsic WNT and BMP4 activation impairs hESC differentiation to definitive endoderm and drives the cells towards extra-embryonic mesoderm. Sci. Rep. *11*, 8242. https://doi.org/10.1038/s41598-021-87547-7.

Martins-Taylor, K., Schroeder, D.I., LaSalle, J.M., Lalande, M., and Xu, R.H. (2012). Role of DNMT3B in the regulation of early neural and neural crest specifiers. Epigenetics *7*, 71–82. https://doi.org/10.4161/epi.7.1.18750.

O'Hara-Wright, M., and Gonzalez-Cordero, A. (2020). Retinal organoids: a window into human retinal development. Development *147*. https://doi.org/10.1242/dev.189746.

Olariu, V., Harrison, N.J., Coca, D., Gokhale, P.J., Baker, D., Billings, S., Kadirkamanathan, V., and Andrews, P.W. (2010). Modeling the evolution of culture-adapted human embryonic stem cells. Stem Cell Res. *4*, 50–56. https://doi.org/10.1016/j.scr.2009.09.001.

Pauklin, S., and Vallier, L. (2015). Activin/Nodal signalling in stem cells. Development *142*, 607–619. https://doi.org/10.1242/dev.091769.

Pera, M.F., and Tam, P.P.L. (2010). Extrinsic regulation of pluripotent stem cells. Nature *465*, 713–720. https://doi.org/10.1038/nature09228.

Price, C.J., Stavish, D., Gokhale, P.J., Stevenson, B.A., Sargeant, S., Lacey, J., Rodriguez, T.A., and Barbaric, I. (2021). Genetically variant human pluripotent stem cells selectively eliminate wild-type counterparts through YAP-mediated cell competition. Dev. Cell *56*, 2455–2470.e10. https://doi.org/10.1016/j.devcel.2021.07.019.

Radeke, M.J., Radeke, C.M., Shih, Y.H., Hu, J., Bok, D., Johnson, L.V., and Coffey, P.J. (2015). Restoration of mesenchymal retinal pigmented epithelial cells by TGFbeta pathway inhibitors: implications for age-related macular degeneration. Genome Med. *7*, 58. https://doi.org/10.1186/s13073-015-0183-x.

Receveur, A., Brisset, S., Martinovic, J., Bazin, A., Lhomann, L., Colmant, C., Pineau, D., Gautier, V., Tosca, L., and Tachdjian, G. (2017). Prenatal diagnosis of isochromosome 20q in a fetus with vertebral anomaly and rocker-bottom feet. Taiwan. J. Obstet. Gynecol. *56*, 677–680. https://doi.org/10.1016/j.tjog.2017.08.018.

Sato, Y., Bando, H., Di Piazza, M., Gowing, G., Herberts, C., Jackman, S., Leoni, G., Libertini, S., MacLachlan, T., McBlane, J.W., et al. (2019). Tumorigenicity assessment of cell therapy products: the need for global consensus and points to consider. Cytotherapy *21*, 1095–1111. https://doi.org/10.1016/j.jcyt.2019.10.001.

Smith, Z.D., Chan, M.M., Mikkelsen, T.S., Gu, H., Gnirke, A., Regev, A., and Meissner, A. (2012). A unique regulatory phase of DNA methylation in the early mammalian embryo. Nature *484*, 339–344. https://doi.org/10.1038/nature10960.

Steinfeld, J., Steinfeld, I., Coronato, N., Hampel, M.L., Layer, P.G., Araki, M., and Vogel-Höpker, A. (2013). RPE specification in the chick is mediated by surface ectoderm-derived BMP and Wnt signalling. Development *140*, 4959–4969. https://doi.org/10.1242/dev.096990.

Taapken, S.M., Nisler, B.S., Newton, M.A., Sampsell-Barron, T.L., Leonhard, K.A., McIntire, E.M., and Montgomery, K.D. (2011). Karotypic abnormalities in human induced pluripotent stem cells and embryonic stem cells. Nat. Biotechnol. *29*, 313–314. https://doi.org/10.1038/nbt.1835.

Thompson, O., von Meyenn, F., Hewitt, Z., Alexander, J., Wood, A., Weightman, R., Gregory, S., Krueger, F., Andrews, S., Barbaric, I., et al. (2020). Low rates of mutation in clinical grade human pluripotent stem cells under different culture conditions. Nat. Commun. *11*, 1528. https://doi.org/10.1038/s41467-020-15271-3.

Vitillo, L., Tovell, V.E., and Coffey, P. (2020). Treatment of age-related macular degeneration with pluripotent stem cell-derived retinal pigment epithelium. Curr. Eye Res. *45*, 361–371. https://doi.org/10.1080/02713683.2019.1691237.

Vugler, A., Carr, A.J., Lawrence, J., Chen, L.L., Burrell, K., Wright, A., Lundh, P., Semo, M., Ahmado, A., Gias, C., et al. (2008). Elucidating the phenomenon of HESC-derived RPE: anatomy of cell genesis, expansion and retinal transplantation. Exp. Neurol. *214*, 347–361. https://doi.org/10.1016/j.expneurol.2008.09.007.

Werbowetski-Ogilvie, T.E., Bossé, M., Stewart, M., Schnerch, A., Ramos-Mejia, V., Rouleau, A., Wynder, T., Smith, M.J., Dingwall, S., Carter, T., et al. (2009). Characterization of human embryonic stem cells with features of neoplastic progression. Nat. Biotechnol. *27*, 91–97. https://doi.org/10.1038/nbt.1516.

Wongtrakoongate, P., Li, J., and Andrews, P.W. (2014). Aza-deoxycytidine induces apoptosis or differentiation via DNMT3B and targets embryonal carcinoma cells but not their differentiated derivatives. Br. J. Cancer *110*, 2131–2138. https://doi.org/10.1038/bjc.2014.128.

Yang, M., Rito, T., Metzger, J., Naftaly, J., Soman, R., Hu, J., Albertini, D.F., Barad, D.H., Brivanlou, A.H., and Gleicher, N. (2021). Depletion of aneuploid cells in human embryos and gastruloids. Nat. Cell Biol. *23*, 314–321. https://doi.org/10.1038/s41556-021-00660-7.

Yu, L., Wei, Y., Duan, J., Schmitz, D.A., Sakurai, M., Wang, L., Wang, K., Zhao, S., Hon, G.C., and Wu, J. (2021). Blastocyst-like structures generated from human pluripotent stem cells. Nature *591*, 620–626. https://doi.org/10.1038/s41586-021-03356-y.

Stem Cell Reports, Volume *18*

Supplemental Information

The isochromosome 20q abnormality of pluripotent cells interrupts germ layer differentiation

Loriana Vitillo, Fabiha Anjum, Zoe Hewitt, Dylan Stavish, Owen Laing, Duncan Baker, Ivana Barbaric, and Pete Coffey

A

Clone	Karyotype
M7 A1	A46,XY [20]
M7 A2	A46,XY [20]
M7 A5	A46,XY,i(20)(q10) [20]
M7 A9	A46,XY,i(20)(q10) [20]
M8 WT	A46,XY [30]
M8 iso20q	A46,XY,i(20)(q10) [20]
M13 WT	A46,XY [30]
M12 iso20q	A46,XY,i(20)(q10) [20]

B

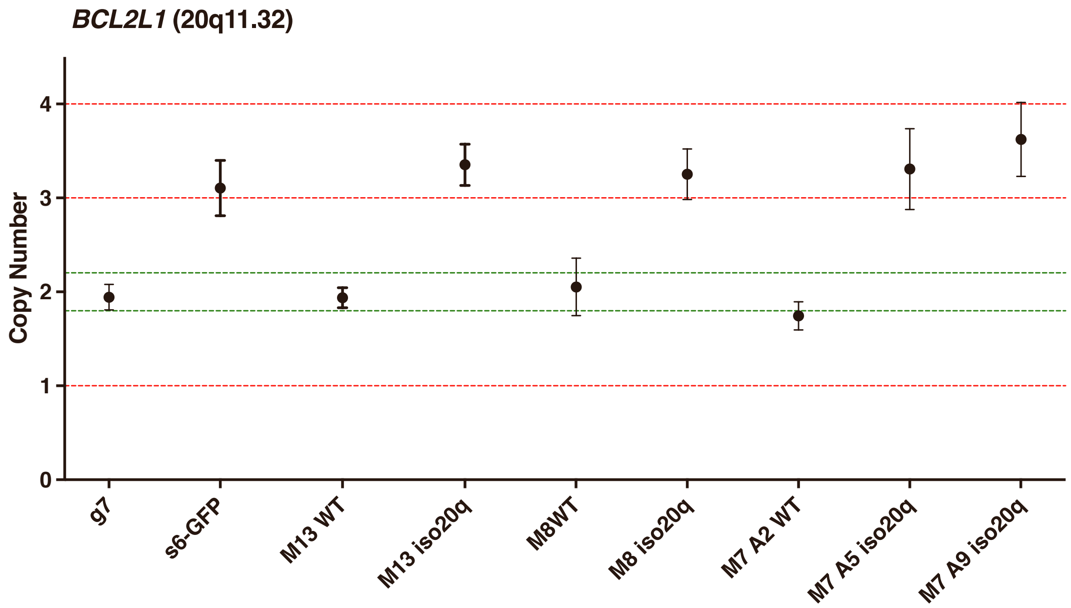

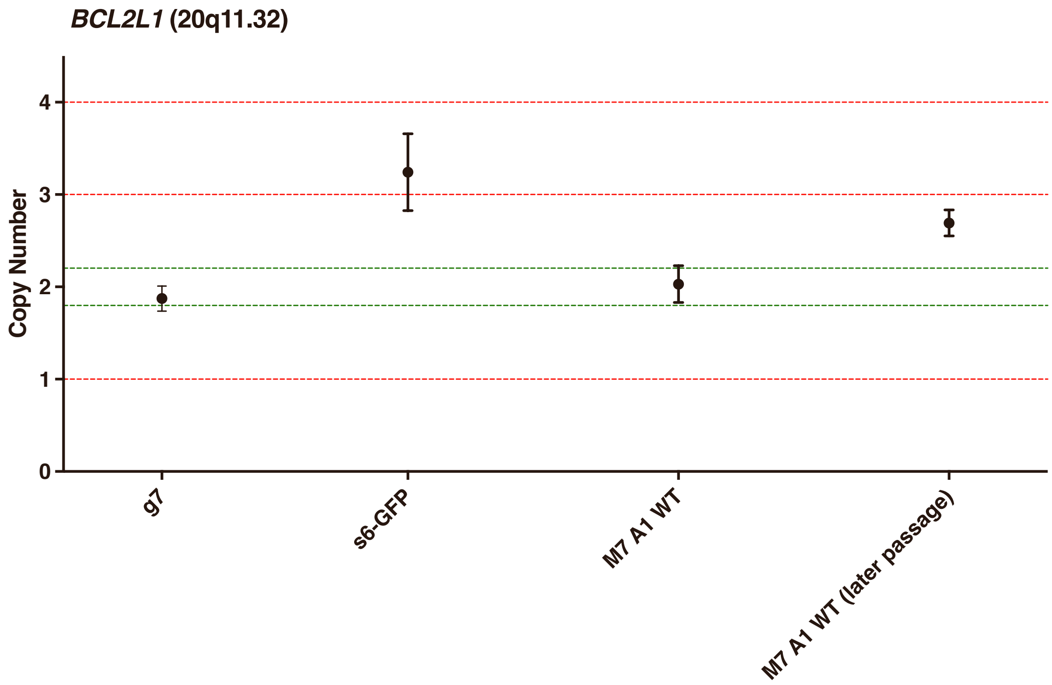

FIGURE S2
Related to FIGURE 2

A

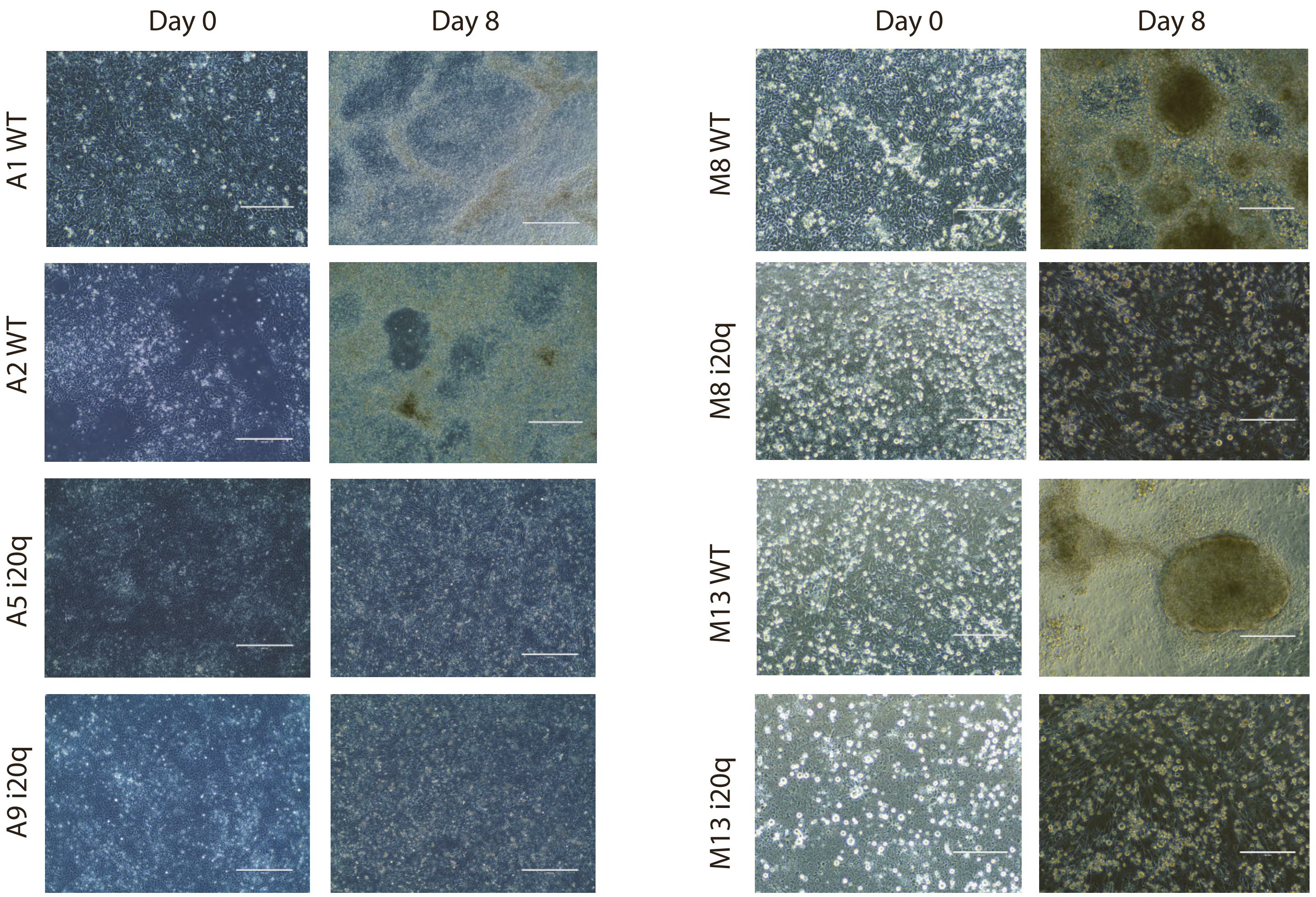

B

Seconday Antibody Control

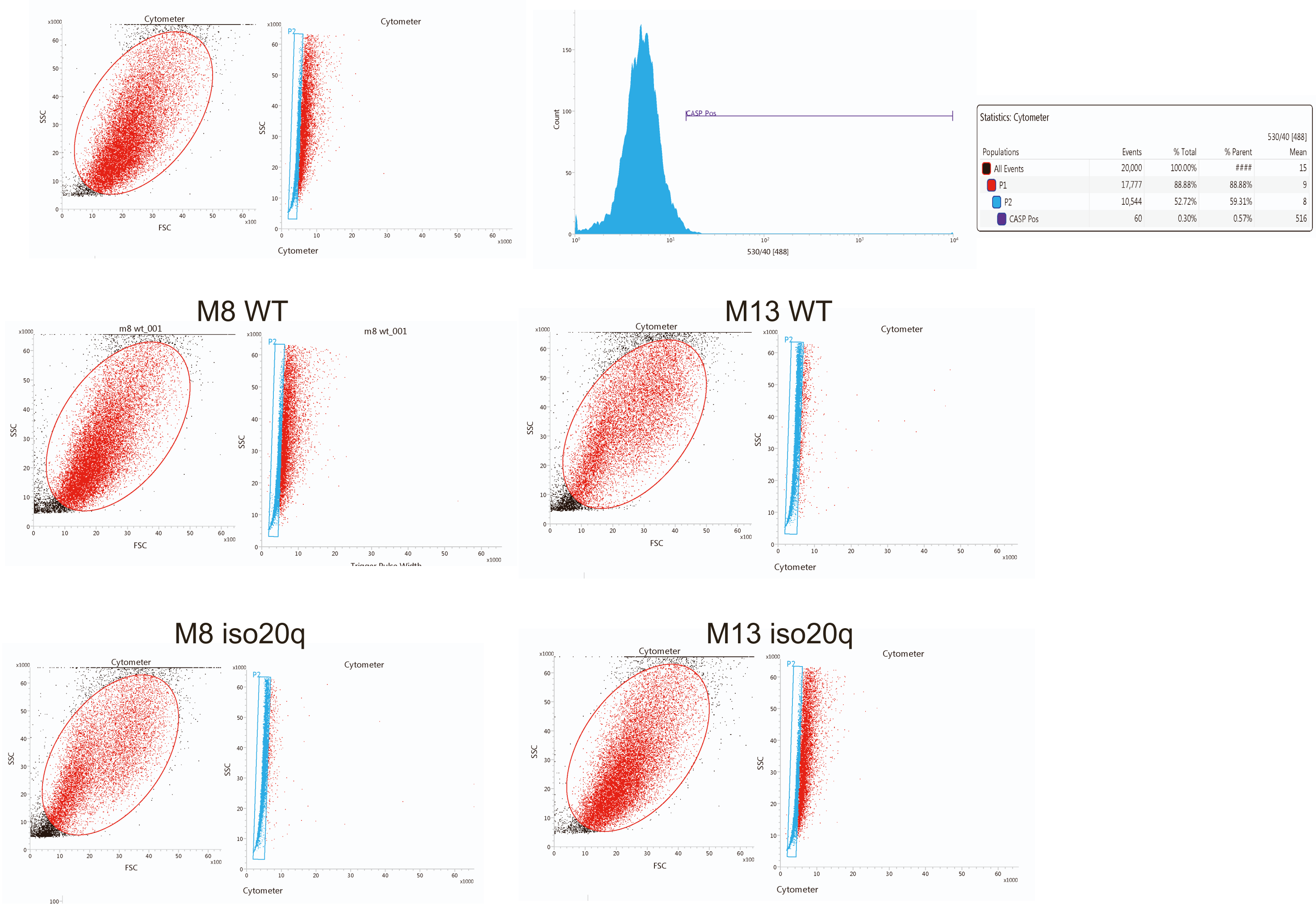

Statistics: Cytometer

Populations	Events	% Total	% Parent	530/40 [488] Mean
All Events	20,000	100.00%	####	15
P1	17,777	88.88%	88.88%	9
P2	10,544	52.72%	59.31%	8
CASP Pos	60	0.30%	0.57%	516

FIGURE S3
Related to FIGURE 3

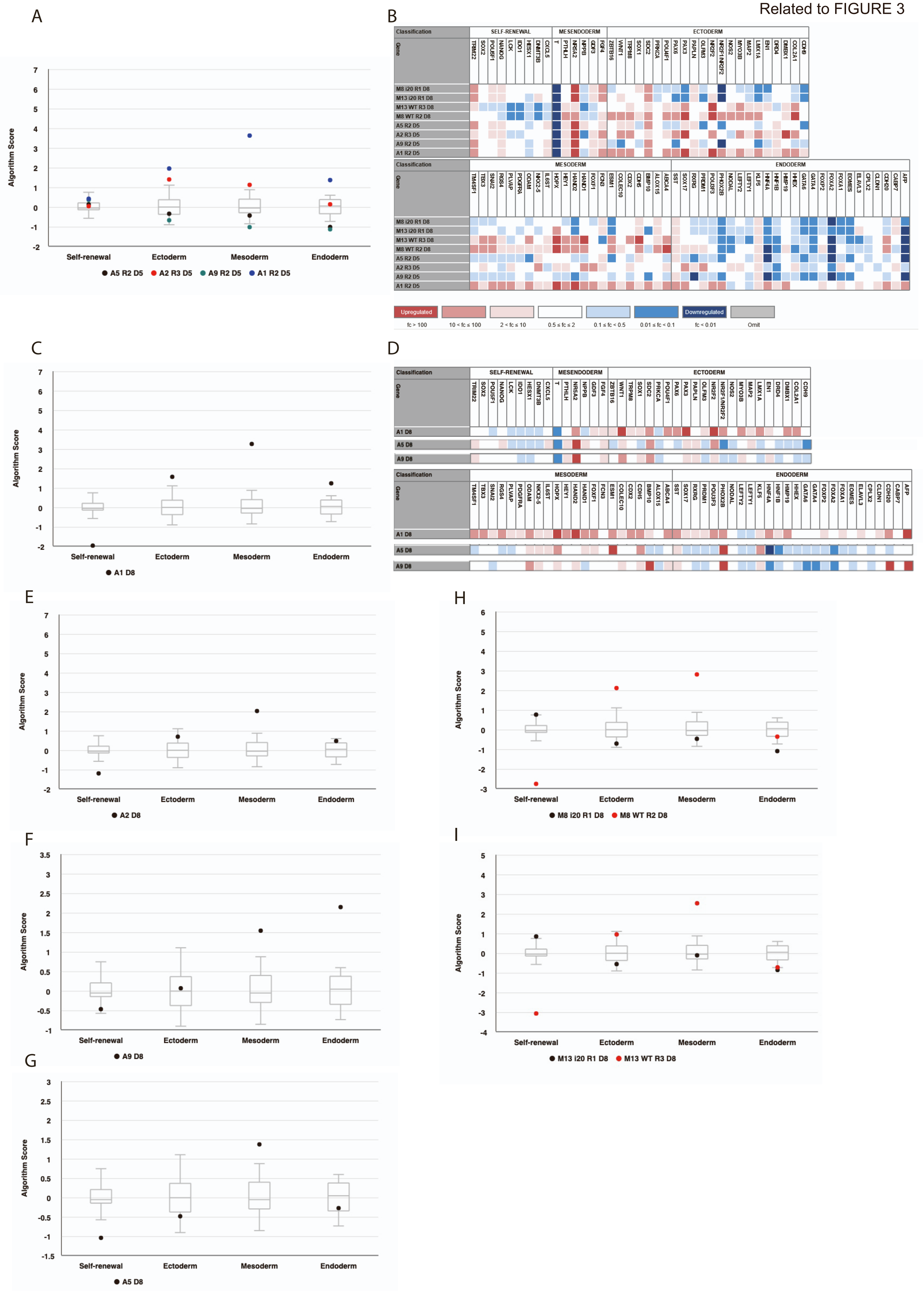

FIGURE S4
Related to FIGURE 3

A

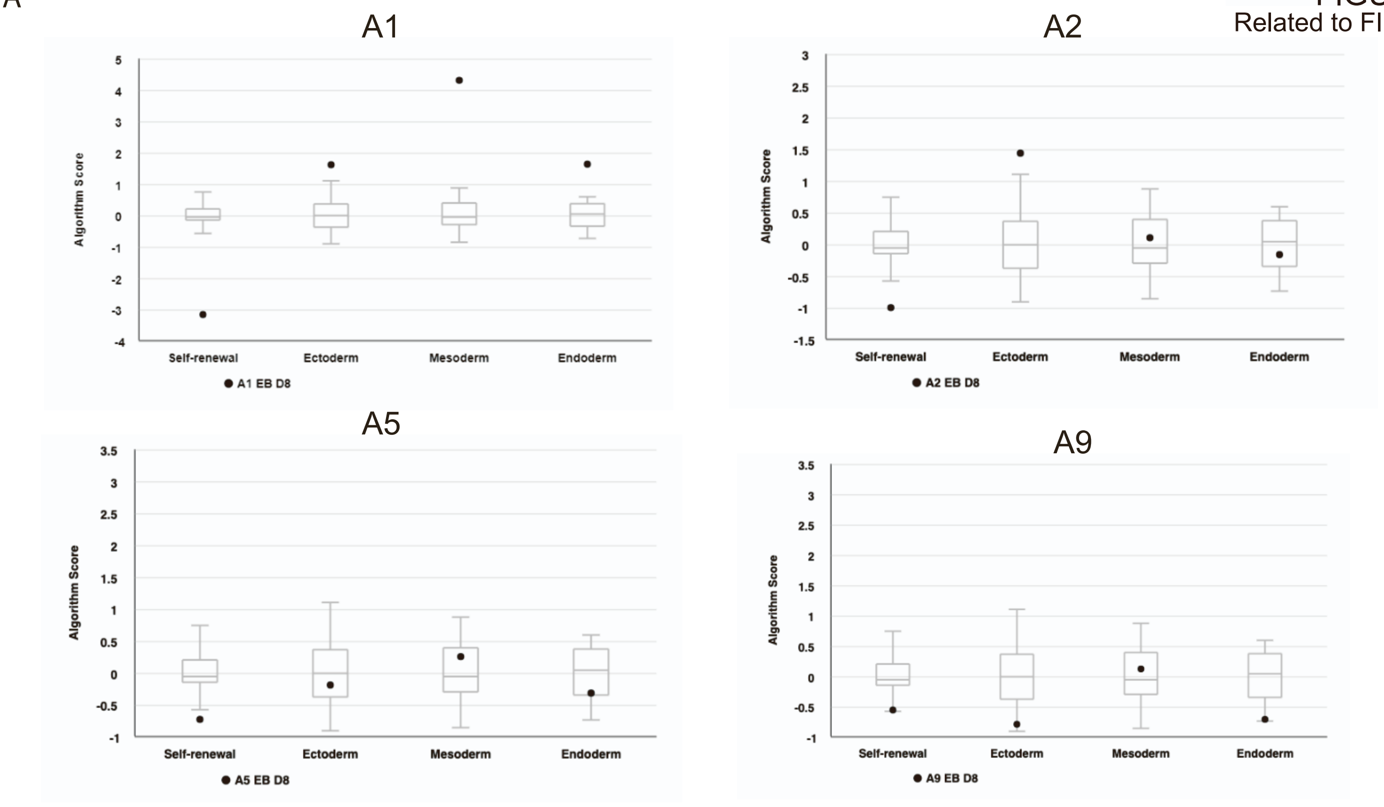

B

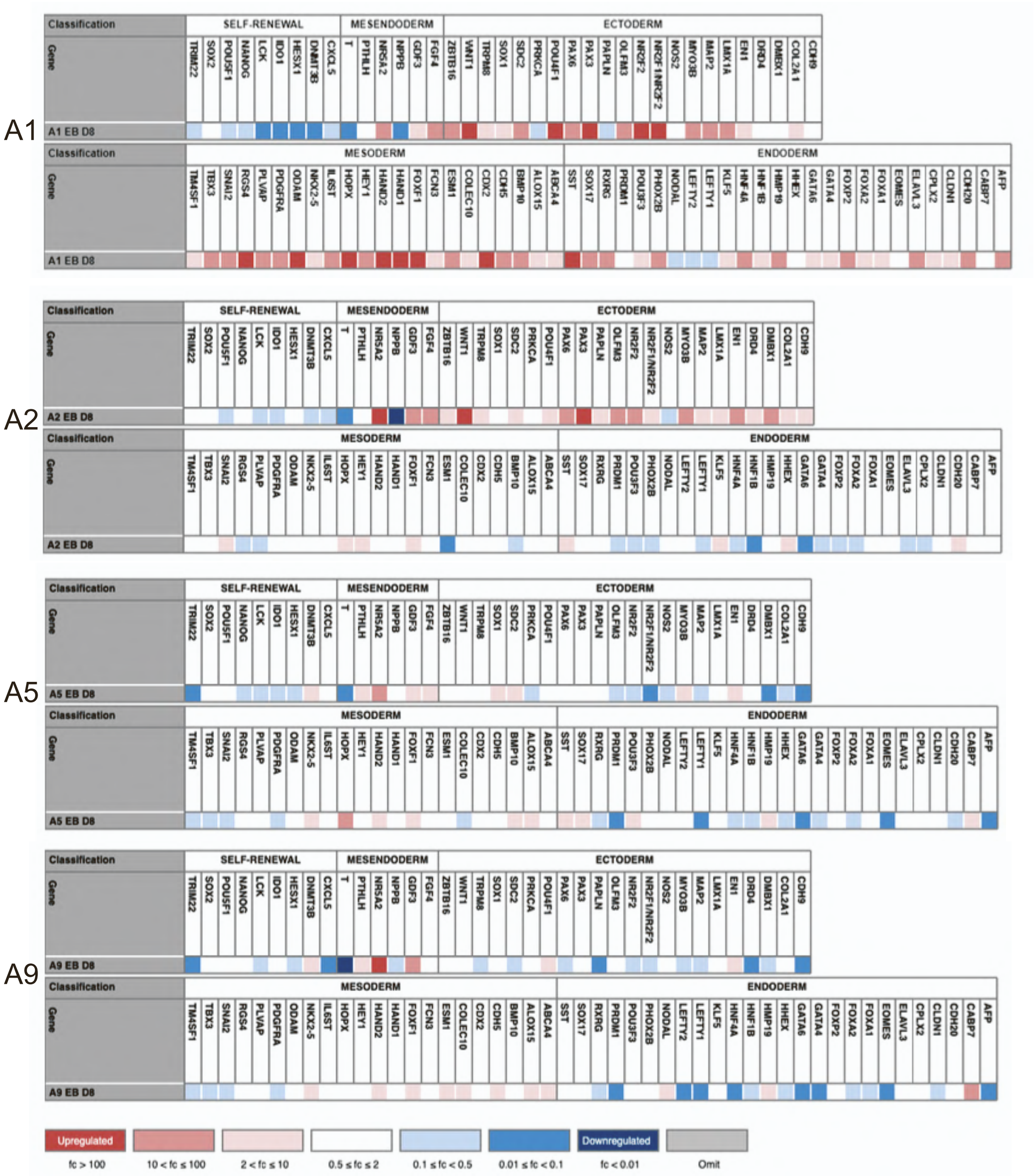

FIGURE S5
Related to FIGURE 4/5

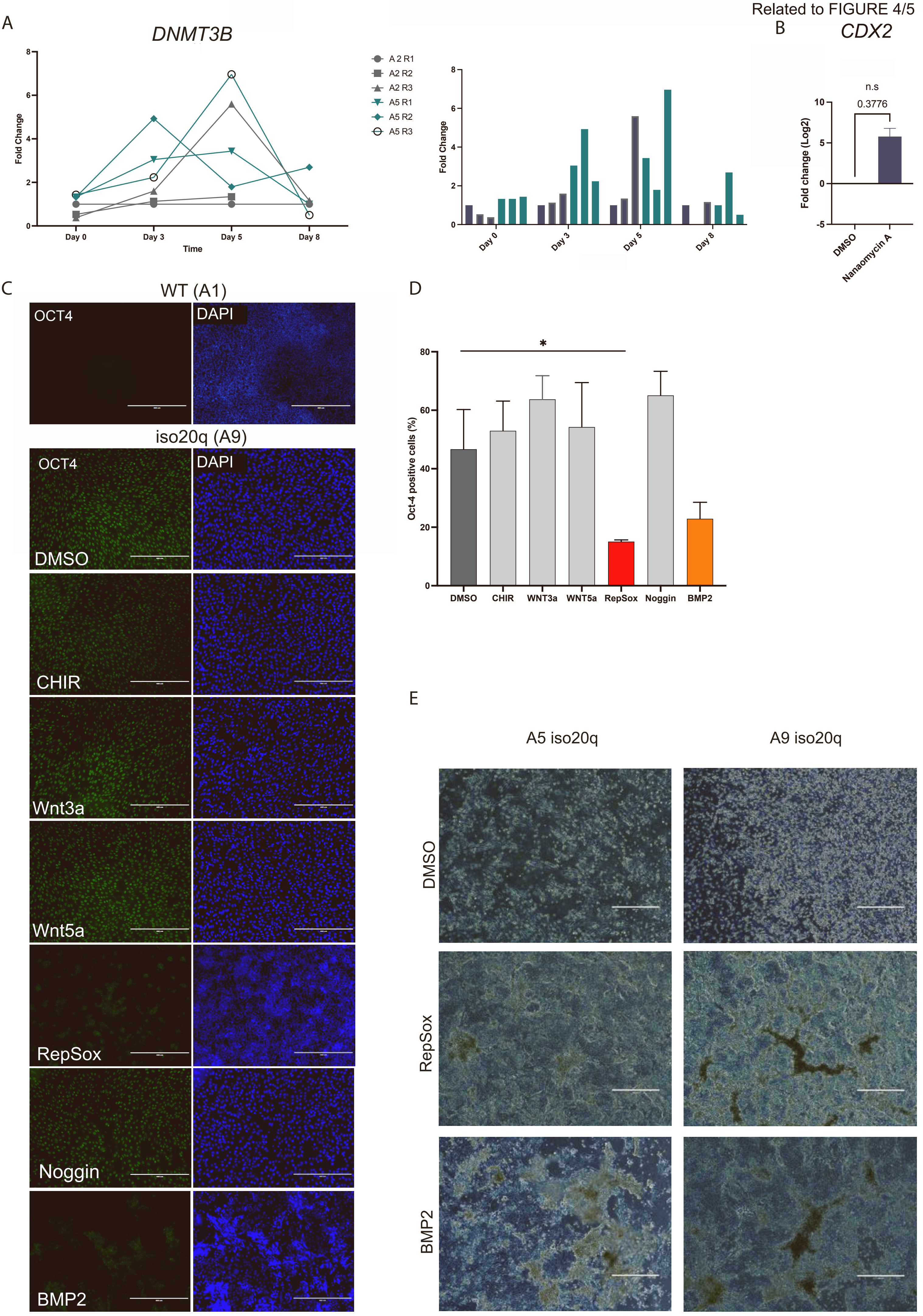

FIGURE S6
Related to FIGURE 6

A

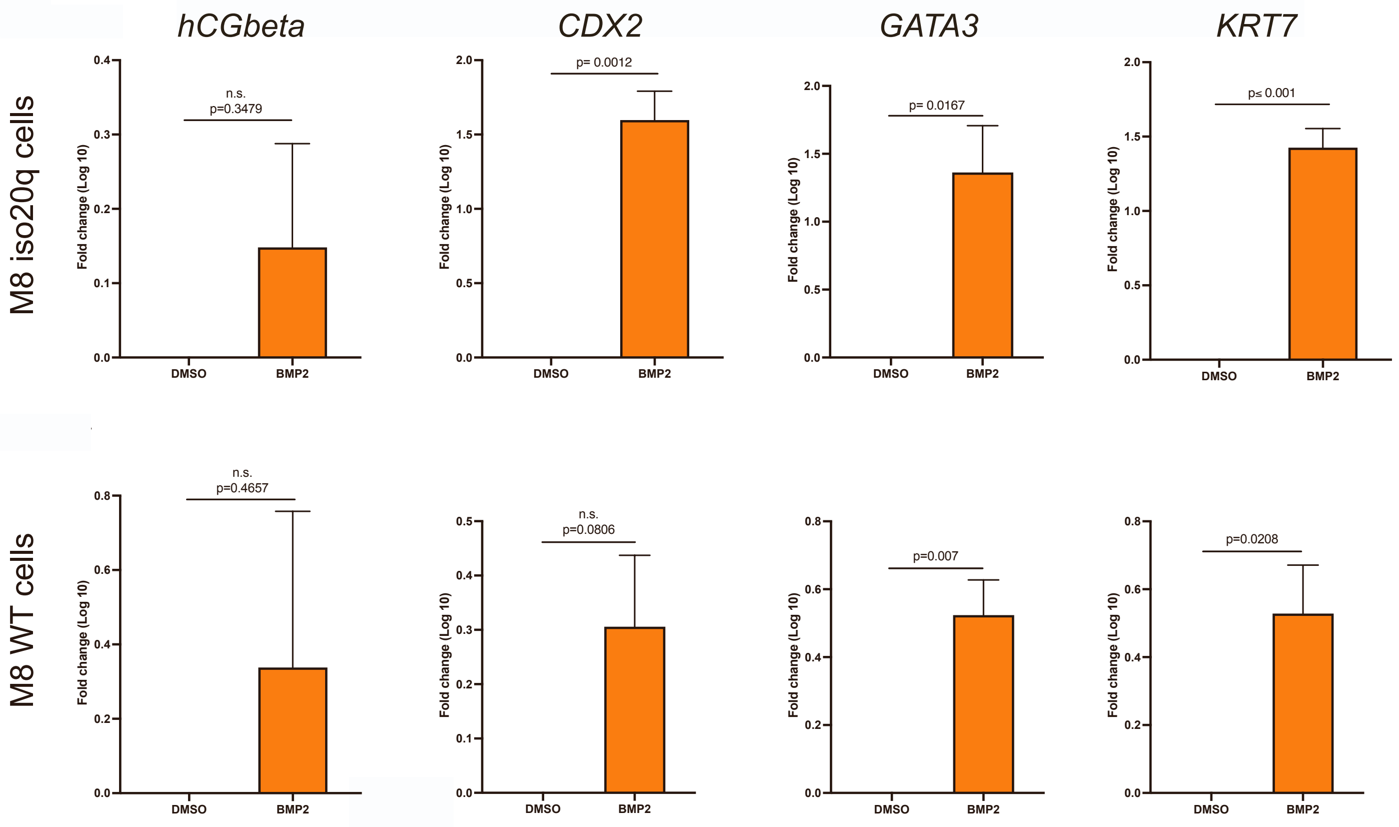

B

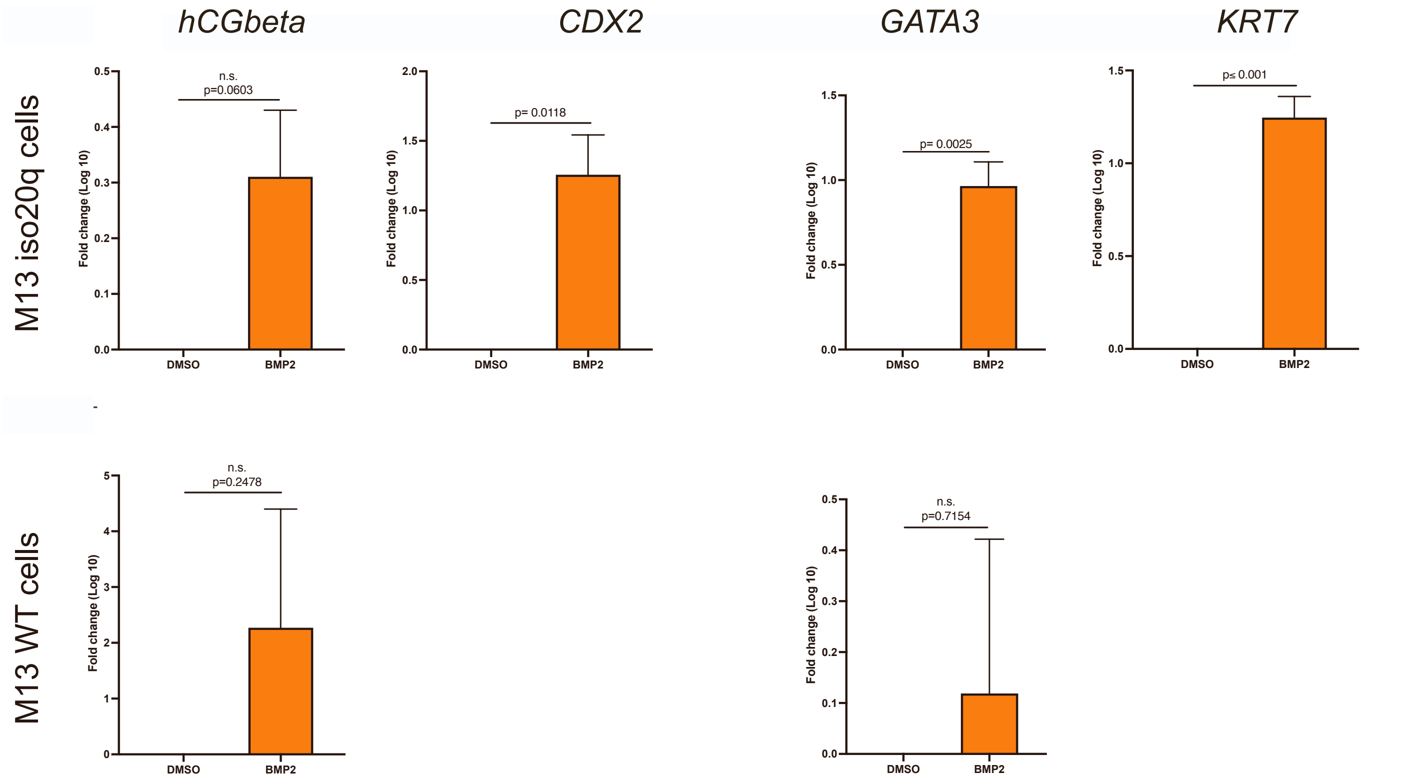

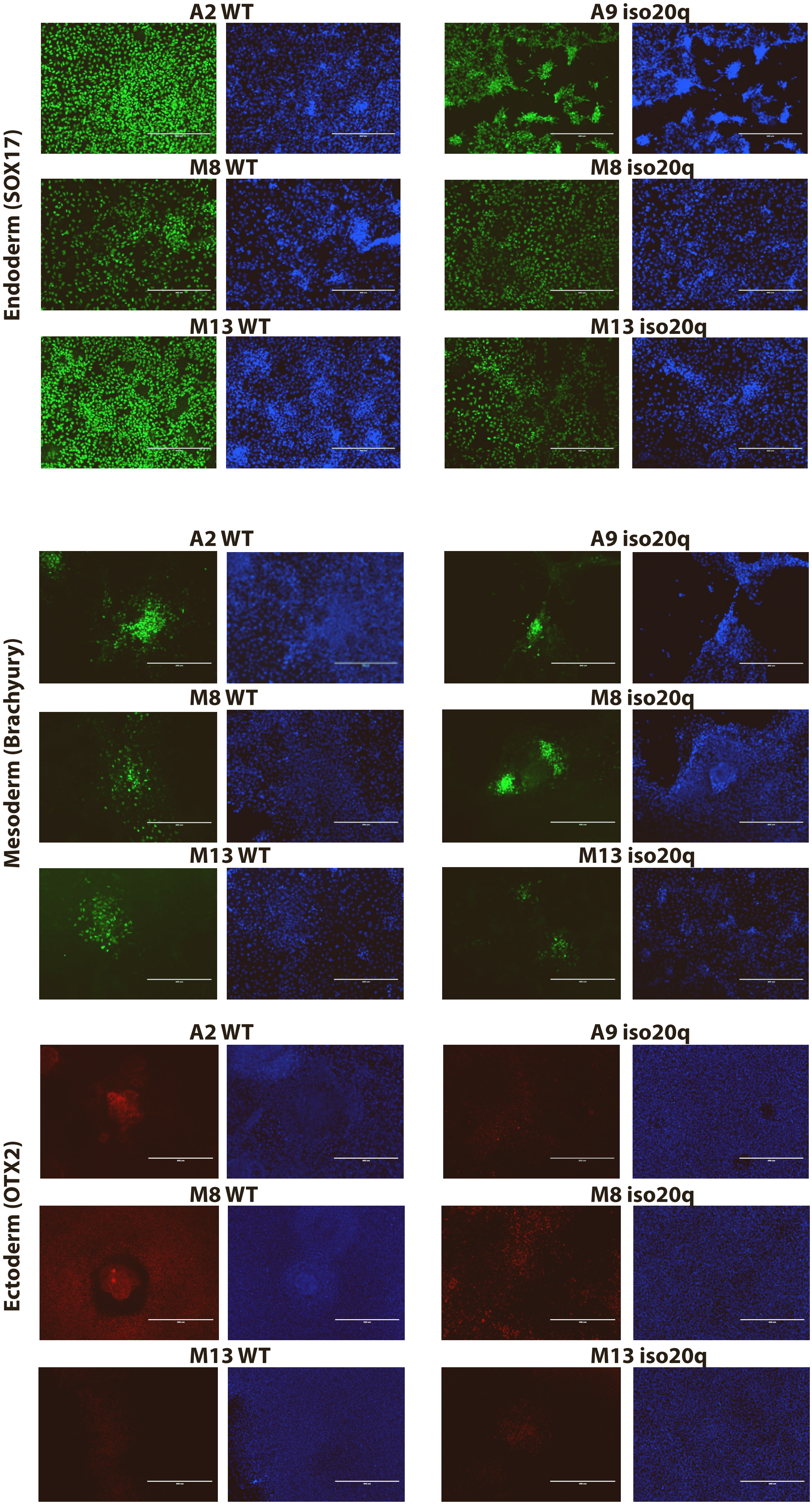
Endoderm (SOX17)
A2 WT
A9 iso20q
M8 WT
M8 iso20q
M13 WT
M13 iso20q
Mesoderm (Brachyury)
A2 WT
A9 iso20q
M8 WT
M8 iso20q
M13 WT
M13 iso20q
Ectoderm (OTX2)
A2 WT
A9 iso20q
M8 WT
M8 iso20q
M13 WT
M13 iso20q

SUPPLEMENTAL FIGURES LEGENDS

Figure S1. Isolating culture acquired chromosome 20 genetic abnormalities.
(Related to Figure 1)

(A) Summary of final quality control karyology for the MasterShef-7, -8, -13 clonal and banked lines.

(B) qPCR copy number assay for BCL2L1 (20q.11.32 locus) in isolated clonal and banked lines. Negative and positive calibrators g7 and s6-GFP are also shown.

Figure S2. Iso20q variants cannot survive a spontaneous RPE differentiation.
(Related to Figure 2)

(A) Representative phase images of iso20q/WT pairs at day 0 and day 8 of RPE differentiation. Scale bars 200 μm.

(B) Gating strategies utilised in caspase-3 flow cytometry analysis in M8 and M13 isogenic lines. Secondary control illustrates the final population of interest (P2) and signal baseline.

Figure S3. The iso20q variant interrupts germ layer differentiation.
(Related to Figure 3)

(A) Algorithm box plot from scorecard qPCR array in A clones at day 5 of differentiation.

(B) Pluripotency scorecard heatmap in iso20q/WT clonal pairs at day 5 (A1, A2, A5, A9 clones) and day 8 (M8, M13 pairs).

(C) Algorithm box plot from scorecard qPCR array in A1 clone at day 8 of differentiation.

(D) Pluripotency scorecard heatmap in iso20q/WT clonal pairs at day 8 (A1, A2, A5, A9 clones).

(E) Algorithm box plot from scorecard qPCR array in A2 clone at day 8 of differentiation.

(F) Algorithm box plot from scorecard qPCR array in A9 clone at day 8 of differentiation.

(G) Algorithm box plot from scorecard qPCR array in A5 clone at day 8 of differentiation.

(H) Algorithm box plot from scorecard qPCR array in M8 WT and iso20q clones at day 8 of differentiation.

(I) Algorithm box plot from scorecard qPCR array in M13 WT and iso20q clones at day 8 of differentiation.

Figure S4. The iso20q variant interrupts germ layer differentiation.
(Related to Figure 3)

(A) Algorithm box plots from scorecard qPCR array in A1, A2, A5, A9 clones at day 8 of EBs formation.

(B) Pluripotency scorecard heatmap in A1, A2, A5, A9 clones at day 8 EBs formation.

Figure S5. DNMT3B-overexpressing iso20q variants are not nullipotent. BMP2 and TGFβ inhibition induce differentiation of iso20q cells.
(Related to Figure 4 and 5)

(A) DNMT3B gene expression in iso20q A5 versus normal A2 clones during 8 days of spontaneous differentiation. R represents an individual experimental run.

(B) Gene expression of trophectoderm marker CDX2 in WT A2 at day 8 of differentiation following treatment with DMSO or nanaomycin A. Data are presented as mean plus SEM, and statistical significance was determined using Student's t test, two-tailed, n=3; ns, not significant.

(C) Representative immunofluorescence images of iso20q A9 clones treated with DMSO or CHIR99021 (1μM), Wnt3A (100 ng/mL), Wnt5A (100 ng/mL), RepSox (100 nM), Nogging (200 ng/mL) or BMP2 (100 ng/mL) for 8 days of spontaneous RPE differentiation and stained with OCT4 and DAPI. The WT A1 clone treated with DMSO is shown as control. Scale bar 400μm.

(D) Quantification of OCT4-positive cells in iso20q A9 clones treated with DMSO or CHIR99021 (1μM), Wnt3A (100 ng/mL), Wnt5A (100 ng/mL), RepSox (100 nM), Nogging (200 ng/mL) or BMP2 (100 ng/mL) for 8 days of RPE differentiation. Data are presented as mean plus SEM, and statistical significance was determined using Student's t test, one-tailed; n=3 independent experiments.

(E) Representative phase images showing the morphological change of A5 and A9 iso20q clones treated with 100 nM RepSox or 100 ng/mL BMP2 versus DMSO for 8 days of RPE differentiation. Scale bars 400μ.

Figure S6. The iso20q variant retain differentiation competency towards extra-embryonic amnion. (Related to Figure 6)

(A) Gene expression of trophoblast markers in M8 iso20q and M8 WT treated with 100 ng/mL BMP2 for 4 days of the RPE differentiation. Data are presented as mean plus SEM, and statistical significance was determined using Student's t test, one-tailed; n=3; n.s. not significant.

(B) Gene expression of trophoblast markers in M13 iso20q and M13 WT treated with 100 ng/mL BMP2 for 4 days of the RPE differentiation. Data are presented as mean plus SEM, and statistical significance was determined using Student's t test, one-tailed; n=3; n.s. not significant.

Figure S7. Directed differentiation protocols mask iso20q underlying developmental defect

(A) Representative immunofluorescence images of iso20q and WT isogenic lines after directed differentiation protocols for endoderm (SOX17), mesoderm (Brachyury) and ectoderm (OTX2). DAPI counterstains are shown in parallel. Scale bars 400µm.

SUPPLEMENTAL EXPERIMENTAL PROCEDURES

Single cell cloning.

Cloning was performed by single cell deposition using a BD FACSJazz cell sorter (BD Biosciences) into 96 well plates with human feeder cells, NutriStem hPSC XF medium (Biological Industries) supplemented with Rock inhibitor Y-27632 (Generon, A11001). After 24 hours the medium was changed to remove Y-27632 and the clones were expanded for 2 weeks before manual picking and culture as described for research cultures. Seed banks were frozen from each clone after 5 passages and material taken for detection of variants by karyotyping and qPCR. Once the genotypes were confirmed the clonal lines were thawed and expanded. MasterShef-7 A1, A2, A5 and A9, were all cloned from the same heterogenous starting population. MasterShef-7, which was derived directly from clinical material but cultured for 8 passages on laminin-521/NutriStem prior to being frozen at passage P5+8. Having indicated a heterogenous i20q karyotype at ~70% prior to freeze, single cell cloning was performed at passage 2 after recovery (P5+8+2) to obtain matched wild type cells from the same culture. Note: a '+' in the passage number refers to a thaw from a cell bank. MasterShef-8 WT (Clone C1.1) was cloned from a homogenous cell bank derived directly from the clinical material but cultured for 5 passage on laminin-521 in NutriStem prior to being frozen P2+9+5 and then subsequently thawed for cloning. The i20q variant (Clone C5.1) was cloned from separate research material which had been culture under its derivation conditions, and then Cellstart/NutriStem before being switched (at P2+9+5+9) to laminin-521/NutriStem culture and frozen as MasterShef-8 QC2.1 at passage P2+9+5+9+19 having acquired the variant which at the point of freeze accounted for ~90% of the cells present. MasterShef-13 WT and i20q lines, were not clonal lines, coming from different research cell banks. Wild type cells came from MasterShef-13 QC1.4 P6+3 derived directly from the clinical material, while MasterShef-13 i20q cells came from bank QC3.1 P6+3+19 cultured on laminin-521.

Genetic copy number q-PCR assay.

Quantitative PCR detection of copy number at loci 20q was performed with our previously described methods and primers[1].

Flow cytometry.

Suspended apoptotic cells were collected together with adherent cells harvested with TrypLE (Thermo Fisher Scientific, 11528856). The collated sample was pelleted and fixed in 4% PFA at room temperature. Flow cytometry for cleaved caspase-3 (Cell Signalling, 9661) was performed with the same methods and reagents we described previously[2]. Samples were analysed using a BD FACSJazz (BD Biosciences). The signal of secondary antibody-only stained samples was used to determine the level of baseline fluorescence. Gating strategies are shown in Figure S2.

Embryoid Bodies.

Confluent hESCs were passaged 1 to 1 into a low-adherence square well plate in 50% Nutristem 50 % TLP media plus 1:1000 Rock inhibitor Y-27632. After 24 hours the newly formed aggregates were transferred to a 15 ml tube to settle and media was fully replaced to TLP. Media was replaced every other day for a week at which point embryoid bodies were collected for RNA extraction.

Immunofluorescence.
Cells were fixed at room temperature for 30 minutes in 4% paraformaldehyde followed by three washes in DPBS. Blocking and permeabilization was performed in DPBS plus 5% Normal Donkey Serum (Jackson Immuno Research Laboratories, 017-000-121) in 0.3% triton X-100 (Sigma). Cells were incubated overnight at 4°C with primary antibodies diluted in 1% donkey serum, 0.3% triton X-100. Next, cells were washed 3 times with DPBS and incubated for 2 hours at room temperature with fluorescent FITC- or TRITC-conjugated donkey secondary antibodies against IgG species matching (Jackson Immuno Research Laboratories) at a dilution of 1:100 in 2% donkey serum, 0-3% triton X-100 (in DPBS). Cells were washed 3 times with DPBS. For nuclei counterstain, cells were incubated for 60 seconds with DAPI (Sigma, D9542, 1:2500) followed by another 3 washes in DPBS. Primary antibodies and dilutions were as follows: goat anti OTX2 (Santa Cruz, 30659, 1:100); rabbit anti OCT-4 (Cell signalling, C30A3, 1:400); goat anti Brachyury (R&D, 967332, 10μg/ml; goat anti SOX17 (R&D, 967330, 10μg/ml). Imaged were acquired using an EVOS FL microscope (Life Technologies) and analysed using Cell Profiler software.

Real-time quantitative PCR.
RNA was extracted from cell pellets using the PureLink RNA mini kit following manufacturer's instruction (Invitrogen). RNA was retrotranscribed to cDNA using the SuperScript III Synthesis System (Invitrogen) or the High capacity cDNA Reverse transcription kit (Applied Biosystems) following manufacturer's instruction. Transcript levels were detected using PowerSYBR™ Green master mix (Applied Byosystems) with StepOne Plus (Applied Biosystems). Gene expression was normalised to GAPDH levels for each sample and final fold change were calculated using the 2-ΔΔCt algorithm. Primers sequence are listed in Table S1.

Western blot array.
Semi-quantitative measurement of 15 stem cell protein markers was performed with the human Stem Cell Array C1 (RayBio®) following manufacturer instructions. Cell pellets were lysed with the supplied lysis buffer supplemented with 1:500 Protease inhibitor cocktail set III (Merk Millipore). Protein extracts were quantified with a Bradford assay (Bio-Rad) and BSA standard curve detected with a Nanodrop™ One (Life Technologies) spectrophotometer. A final amount of 250 μg was loaded onto each protein array. Blots were scanned with Chemidoc (Bio-Rad). Densitometries were extracted from scanned high-resolution images with ImageJ software. Results were analysed according to manufacturer instruction considering averages of target signals relative to internal positive controls and normalised to reference array (DMSO array).

Small molecules and morphogens.
DNMT3b inhibitor Nanaomycin A (BioVision, 2790-1), CHIR990-21 (Miltenyi, StemMACS™), recombinant human Wnt3a (R&D, 5036-WN-010), recombinant human/mouse Wnt5a (R&D, 645-WN-010), RepSox, recombinant human Noggin (R&D, 6057-NG-025), recombinant human/mouse/rat BMP2 (R&D, 355-BM-010), DMSO (Sigma, 02438).

Directed differentiation.
Directed trilineage differentiation were performed with standardised StemXVivo Endoderm (R&D, SC019B), StemXVivo Mesoderm (R&D, SC030B) and STEMdiff™ Trilineage Ectoderm Medium (05231) kits, following manufacturer's instruction. Endoderm and Mesoderm differentiations were stopped at day 4 while ectoderm differentiation was stopped at day 7. For these experiments cells were expanded in mTERS1 (Stem Cell Technologies, 85850) and plated on Cultrex Basement Membrane Extract (R&D, 3432-005-01) for Endoderm and Mesoderm differentiation or Matrix Matrigel hESc qualified (Corning, 734-1440) for Ectoderm differentiation.

Table S1. Primers set.

Gene target	Forward	Reverse
DNMT3B	CTGCCGGTGTTTCTGTGTGG	TGTAACAGCTCCAGGGCTCC
BCL-XL	CTGCCGGTGTTTCTGTGTGG	TCCACAAAAGTATCCCAGCC
OTX2	GCGCAGCTAGATGTGCTGGA	CACTGCTGCTGGCAATGGTC
PAX6	CCATCAGACCCAGGGCAATC	GGTCTGCCCGTTCAACATCC
CDX2	TTCACTACAGTCGCTACATCACC	TTGATTTTCCTCTCCTTTGCTC
hCGB	ACCCTGGCTGTGGAGAAGG	ATGGACTCGAAGCGCACA
GATA3	TGCAGGAGCAGTATCATGAAGCCT	GCATCAAACAACTGTGGCCAGTGA
KRT7	AGGATGTGGATGCTGCCTAC	CACCACAGATGTGTCGGAGA
GAPDH	CCCCACCACACTGAATCTCC	GGTACTTTATTGATGGTACATGACAAG
	**Reference number (Thermo Fisher Scientific)**	
SOX17	HS00751752_s1	
T	Hs00610080_m1	

SUPPLEMENTAL REFERENCES

1) Laing, O., Halliwell, J., and Barbaric, I. (2019). Rapid PCR Assay for Detecting Common Genetic Variants Arising in Human Pluripotent Stem Cell Cultures. Curr Protoc Stem Cell Biol 49, e83. 10.1002/cpsc.83.

2) Price, C.J., Stavish, D., Gokhale, P.J., Stevenson, B.A., Sargeant, S., Lacey, J., Rodriguez, T.A., and Barbaric, I. (2021). Genetically variant human pluripotent stem cells selectively eliminate wild-type counterparts through YAP-mediated cell competition. Dev Cell 56, 2455-2470 e2410. 10.1016/j.devcel.2021.07.019.
